# Clinical characteristics and outcomes of diabetes‐related ketoacidosis (DKA) in sodium‐glucose co‐transporter‐2 inhibitor (SGLT2i) users with type 2 diabetes

**DOI:** 10.1111/dom.70098

**Published:** 2025-09-08

**Authors:** Angelica Sharma, Shams Ali Baig, Rasiah Thayakaran, Lakshmi Rengarajan, Nevil C. Philip, Anu Ann Abraham, Aspasia Manta, Parth Narendran, Ketan Dhatariya, Guillermo E. Umpierrez, Punith Kempegowda, DEVI collaboration

**Affiliations:** ^1^ Department of Endocrinology Norfolk and Norwich University Hospitals NHS Foundation Trust Norwich UK; ^2^ Department of Applied Health Sciences University of Birmingham Birmingham UK; ^3^ Birmingham Medical School University of Birmingham Birmingham UK; ^4^ Queen Elizabeth Hospital University Hospitals Birmingham NHS Foundation Trust Birmingham UK; ^5^ Institute of Immunology and Immunotherapy University of Birmingham Birmingham UK; ^6^ Norwich Medical School University of East Anglia Norwich UK; ^7^ Division of Endocrinology and Metabolism Emory University School of Medicine Atlanta Georgia USA

**Keywords:** acute complications, diabetes ketoacidosis, sodium‐glucose co‐transporter‐2 inhibitors, type 2 diabetes

## Abstract

**Aim:**

Sodium‐glucose co‐transporter‐2 inhibitors (SGLT2i) offer significant cardiorenal benefits for people with type 2 diabetes (PwT2D). However, concerns remain regarding their association with diabetes‐related ketoacidosis (DKA). (1) To compare demographics, precipitating factors, biochemical features, management, and outcomes of acute DKA admissions between SGLT2i users (*n* = 267) and non‐users (*n* = 793) with T2D. (2) To conduct a systematic review and meta‐summary of published studies describing SGLT2i‐associated DKA in T2D.

**Methods:**

A retrospective cohort study analysed data from 18 UK hospitals (April 2018–March 2024), using standardised DKA protocols. Propensity score matching compared DKA episodes between SGLT2i users and non‐users. In addition, a systematic review and meta‐summary was performed including studies from PubMed, EMBASE, MEDLINE, Scopus, and Web of Science focusing on DKA in PwT2D treated with SGLT2i.

**Results:**

Within the DEKODE cohort, 534 matched individuals were analysed. SGLT2i users had lower glucose, pH, and bicarbonate levels than non‐users. SGLT2i was identified as the sole precipitant in 30.3% of cases. Despite lower admission glucose and more profound acidosis, both SGLT2i users and non‐users had similar clinical outcomes including duration of DKA and length of hospital stay. In the meta‐summary of 1024 cases of SGLT2 inhibitor‐associated DKA from 247 studies, the median age was 54.6 years, with 49.7% male and a median diabetes duration of 10 years. Biochemical features included acidosis (median pH 7.1), elevated ketones (5.7 mmol/L), and modest hyperglycaemia (10.6 mmol/L). DKA typically developed after 2 months of SGLT2i use, with 21.1% requiring intensive care admission.

**Conclusions:**

Despite lower admission glucose, more pronounced acidosis, and a higher incidence of hypokalaemia episodes, clinical outcomes were similar between the matched population of SGLT2i users and non‐users. This may be attributed to earlier identification of euglycaemic DKA, timely intervention, as well as the distinct pathophysiological profile of SGLT2i‐associated DKA. Improved education on risk factors and preventive strategies is warranted with SGLT2i therapy.

## 1 INTRODUCTION

Sodium‐glucose co‐transporter‐2 inhibitors (SGLT2i) have gained popularity due to favourable outcomes reported in cardiovascular outcome trials.^1^, ^2^, ^3^, ^4^, ^5^ Additionally, landmark trials such as CREDENCE, DAPA‐CKD, and EMPA‐KIDNEY have shown reduced progression of chronic kidney disease (CKD) with the use of SGLT2i.^6^ The American Diabetes Association's ‘Standards of Care in Diabetes’ recommend using SGLT2i in people with type 2 diabetes and either established atherosclerotic cardiovascular disease (ASCVD), high ASCVD risk, heart failure (HF), or chronic kidney disease.^7^ Similarly, the updated National Institute for Health and Care Excellence guidelines in the UK advocate for dual first‐line therapy with metformin and SGLT2i therapy for individuals with established ASCVD or HF and recommend considering SGLT2‐inhibitor therapy for those at high risk of cardiovascular disease or with an elevated lifetime risk of CVD.^8^

Despite these benefits, concerns have been raised regarding the safety profile of SGLT2i.^9^, ^10^ SGLT2i promote glycosuria by shunting glucose into the urine, leading to a shift in energy metabolism towards lipid oxidation and increased ketone production. This is mediated by a reduction in the insulin‐to‐glucagon ratio, which favours ketogenesis.^11^ Mild to moderate ketonemia is therefore a common physiological effect, often detectable within days of initiating therapy.^12^ This metabolic profile may explain the increased susceptibility to DKA observed in patients treated with SGLT2i compared to those on other glucose‐lowering therapies.

Previous meta‐analyses have reported conflicting findings on the increased risk of DKA associated with SGLT2i.^13^, ^14^, ^15^, ^16^ These meta‐analyses and randomised controlled trials do not provide detailed information on the clinical characteristics and individualised patient profiles of people with type 2 diabetes receiving SGLT2i who develop DKA. Additionally, the limited number of events constrains the generalisability of these findings. Existing real‐world studies on SGLT2i‐associated DKA have primarily been confined to small‐scale case reports or series, often originating from single centres with short study durations.^17^, ^18^, ^19^, ^20^, ^21^ These studies demonstrate heterogeneous clinical outcomes, limiting meaningful comparisons and interpretations. By aggregating data from multiple studies, we aim to clarify the clinical features and outcomes of affected individuals, contributing to safer and more effective diabetes management. However, a key limitation remains: the variation in management protocols and guidelines across the studies.

The Digital Evaluation of Ketosis and Other Diabetes Emergencies (DEKODE) initiative is a multi‐centre collaboration in the UK that analyses DKA admissions, management strategies, and outcomes across 28 hospitals.^22^, ^23^ All participating hospitals follow an integrated care pathway for DKA management based on the Joint British Diabetes Societies for Inpatient Care guidelines.^24^ DEKODE, therefore, provides an opportunity to study the natural history of DKA episodes managed similarly and their impact on patient outcomes. Using data from the DEKODE study, we conducted a retrospective cohort analysis to examine whether differences exist in the presentation and outcomes of DKA between SGLT2i users and non‐users with type 2 diabetes managed under similar protocols.

## 2 METHODS

### 2.1 Systematic review and meta‐summary

*Search strategy*: A systematic review was conducted in accordance with PRISMA guidelines to identify studies on DKA in people with type 2 diabetes treated with SGLT2i. Searches were performed across PubMed, EMBASE, MEDLINE, Scopus, and Web of Science without publication year. Keywords included ‘diabetic ketoacidosis’, ‘SGLT2 inhibitors’, and specific drug names (e.g., canagliflozin, dapagliflozin, and empagliflozin), with the full search strategy detailed in Supplementary Material 1. Boolean operators were used to maximise retrieval. Two independent authors screened abstracts and full texts using COVIDENCE software.

*Inclusion*/*exclusion criteria*: Eligible studies included case reports, case series, observational and retrospective cohort studies published in English that focused on real‐world clinical data. Conference abstracts, studies involving people without type 2 diabetes, patients under 16 years of age, and with missing clinical DKA data were excluded. The PRISMA flow diagram (Supplementary Material 2) details the study selection process for this systematic review and meta‐summary.

*Data extraction*: Standardised data extraction captured demographics (age, gender, ethnicity, and BMI), clinical characteristics (duration of diabetes, most recent HbA1c levels, duration of SGLT2i), biochemical parameters, management, and outcomes, including critical care admissions, haemodialysis, hypoglycaemia, and hypokalaemia. Two independent reviewers resolved discrepancies through discussion or by consulting a third reviewer. Data were compiled in SPSS for analysis.

*Statistics*: Parameters are reported using descriptive statistics as (%, *n*) for categorical data and as median (interquartile range (IQR)) for continuous data. For *n* = 3 or fewer samples, data are reported as median (range) and as a single value for samples where *n* = 1. In the systematic review, source studies reported continuous variables using a variety of summary statistics, including means, medians, and ranges. To enable consistent reporting across heterogeneous datasets, we applied established conversion methods to estimate medians and IQRs from the reported values.^25^ Owing to the lack of access to raw individual‐level data, formal testing for normality across studies was not feasible. Therefore, to reduce the influence of skewed distributions and maintain comparability, all continuous variables in the meta‐summary were presented as median (IQR). For parameters with missing data, the number of studies contributing to the statistical analyses is specified. The statistical analyses were conducted using SPSS (version 29.0, IBM SPSS Inc., Chicago, IL, United States).

### 2.2 DEKODE cohort data

**FIGURE 1:**
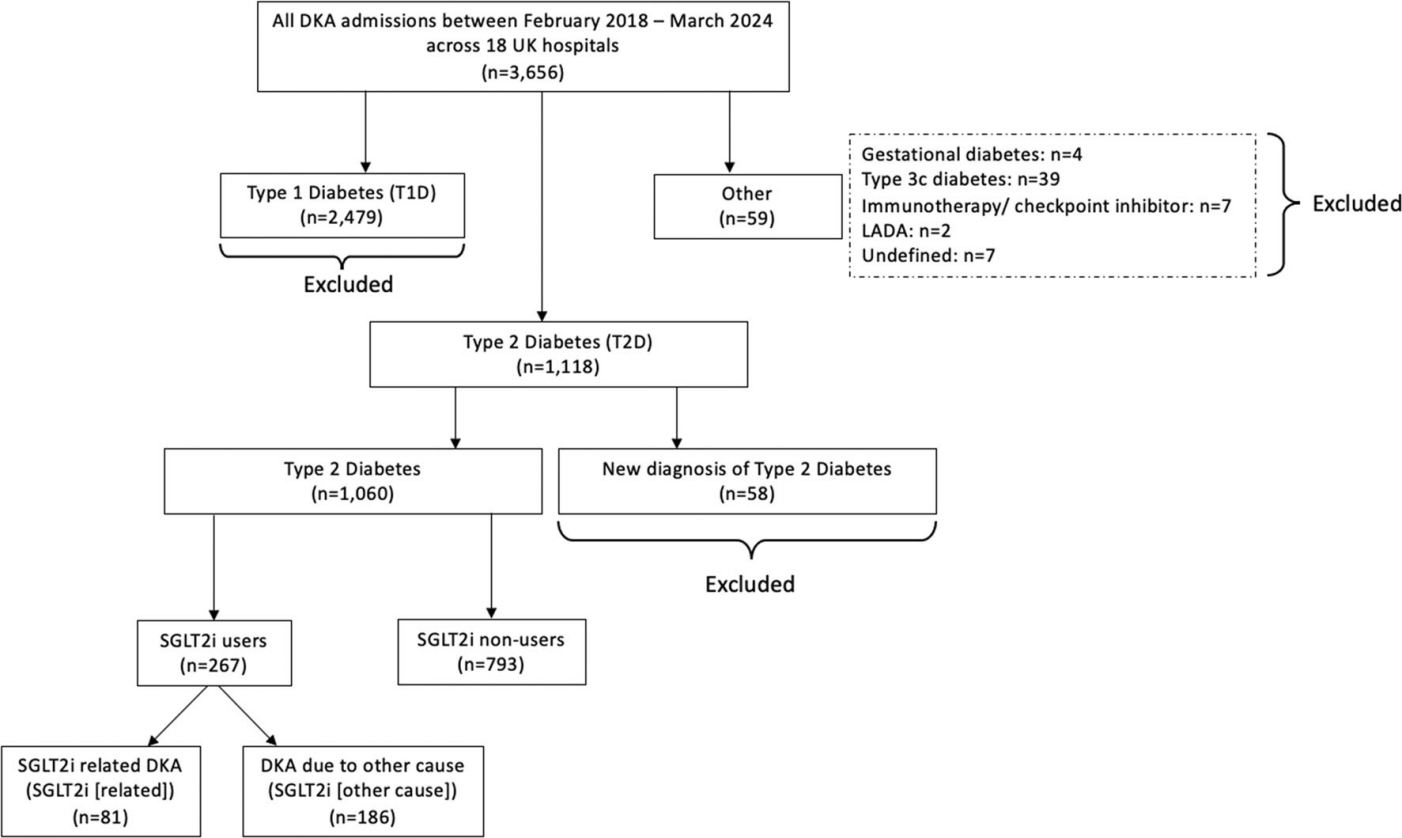
Flowchart of DKA case selection from the DEKODE database for retrospective cohort study.

From 3656 DKA episodes in the DEKODE database (April 2018–March 2024), cases involving type 1 diabetes (*n* = 2479), newly diagnosed diabetes at the time of DKA (*n* = 58), and other forms of diabetes (*n* = 59) were excluded. The final cohort consisted of 1060 DKA cases in people with type 2 diabetes, divided into SGLT2i users (*n* = 267) and non‐users (*n* = 793). Among SGLT2i users, cases were categorised as either SGLT2i‐related (*n* = 81) or those with precipitating causes (*n* = 186), based on the presenting history, clinical examination, biochemistry data, and clinical judgement of the treating consultant physician (Figure 1).

*Key outcomes*: Differences in DKA precipitants, biochemical values at admission, and clinical outcomes (i.e., hypoglycaemia, electrolyte imbalances, DKA duration, length of hospital stay, intensive care unit (ICU) admission, and mortality) were assessed.

*Statistical analysis*: R software (v4.3.0) was used for all analysis. The Matchit package within R was used for performing propensity score matching. Categorical variables (nominal or ordinal) were reported as frequencies with percentages, while continuous variables were measured by mean ± standard deviation (SD) or median with IQR. Normality was assessed using both visual and statistical approaches. Specifically, we examined histograms and Q–Q plots for each continuous variable and performed the Shapiro–Wilk test to formally evaluate the normality assumption. Variables with *p*‐values less than 0.05 were considered non‐normally distributed and were analysed using appropriate non‐parametric methods accordingly.

The unpaired t test was utilised if both variables (with subjects in both groups being independent of each other) were normally distributed and continuous. Conversely, the Mann–Whitney U test was employed to compare two unpaired, non‐normally distributed variables. The Chi‐square test was used for non‐parametric categorical data, and Fisher's exact test was applied for non‐parametric categorical variables with low frequencies (<5).

Propensity score matching (1:1) was employed to reduce confounding and balance baseline characteristics between SGLT2i users and non‐users when comparing episodes of DKA. The propensity score represents the probability of being prescribed an SGLT2i, estimated for each patient using a logistic regression model based on relevant covariates such as age, gender, BMI, ethnicity and CCI categories. After calculating the propensity scores, each SGLT2i user was matched to one or more non‐user(s) with similar scores, typically using nearest neighbour matching without replacement and a calliper to limit acceptable differences in scores. This matching process aimed to create comparable groups with similar distributions of observed covariates, minimising selection bias. After matching, the balance of baseline characteristics was assessed using standardised mean differences to ensure adequate comparability. DKA episodes were then compared between the matched SGLT2i user and non‐user cohorts, allowing for a less confounded estimation of the association between SGLT2i use and DKA risk.

Subgroup analyses included comparisons of SGLT2i‐related DKA to cases with identifiable precipitating factors. In further subgroup analysis, individuals on SGLT2i without another identifiable precipitating cause of DKA [SGLT2i‐related] were matched 1:1 with SGLT2i users with identifiable precipitating factors [SGLT2i with precipitating cause]. All DKA episodes were classified by the presenting history, clinical examination, and biochemical values at the final discretion of the treating consultant physician. In addition, SGLT2i non‐users and those who developed DKA due to another cause whilst on concomitant SGLT2i therapy [SGLT2i (with precipitating cause)] were matched 1:1 to compare clinical profiles. Missing data were handled using multiple imputations with the *mice* R package. Statistical significance was set at *p* <0.05.

### 2.3 Ethical considerations

The data was obtained following approval from information governance at all hospitals in the DEKODE database at the time of data extraction (approval numbers: 12074; 1538; 16468; 6118; 11539; Diab/QI/2022‐23/08; 2499; QIP 010; 11 713; DIAB‐22‐23‐A08; QI20‐21/LTC/01; CA‐2023‐24‐062). All data were pseudonymised at the point of collection.

## 3 RESULTS

### 3.1 Systematic Review and Meta‐Summary

We identified 1024 people with type 2 diabetes from 247 studies with SGLT2 inhibitor‐associated DKA. The findings are summarised in Table 1, Supplementary Material 3 with a summary of references in Supplementary Material 4. The median (IQR) age was 54.6 (45.0–61.7) years, with 49.7% being male and a median BMI of 28.9 (24.3–33.4) kg/m^2^. Individuals had lived with type 2 diabetes for a median duration of 10 (6.0–14.8) years and had a median admission HbA1C of 77.5 (63.5–89.3) mmol/mol (9.2% (8.0–10.3)). Within the meta‐summary, key precipitants included SGLT2i use itself (26.9% for empagliflozin, 23.5% for dapagliflozin, and 20.1% for canagliflozin), intercurrent illnesses, and surgical admissions (Supplementary Material 5).

**TABLE 1:** Meta‐summary of SGLT2i‐related DKA episodes [1–247].

Parameters	Total population (studies [*s*] = 247, [*n*] = 1024)	Asia^1–80^ (studies [*s*] = 80, [*n*] = 269)	Europe^81–119^ (studies [*s*] = 39, [*n*] = 146)	North America^120–227^ (studies [*s*] = 108, [*n*] = 299)	Oceania^228–243^ (studies [*s*] = 16, [*n*] = 305)	South America^244–247^ (studies [*s*] = 4, [*n*] = 5)
Sample size	*N* = 1024	*N* = 269	*N* = 146	*N* = 299	*N* = 305	*N* = 5
Age, years	54.6 (45.0–61.7) [*s* = 246]	55.7 (45.0–61.0) [*s* = 78]	57.0 (49.0–63.0) [*s* = 39]	52.0 (43.0–59.3) [*s* = 107]	62.1 (54.4–63.8) [*s* = 16]	59.0 (51.5–75.5) [*s* = 4]
Gender (male), % [*n*]	49.7% (*n* = 509/1024)	46.8% (*n* = 126/269)	45.9% (*n* = 67/146)	53.2% (*n* = 159/299)	50.5% (*n* = 154/305)	60% (*n* = 3/5)
Ethnicity						
White	4.7% (*n* = 48/1024)	0.4% (*n* = 1/269)	2.7% (*n* = 4/146)	5.4% (*n* = 16/299)	8.9% (*n* = 27/305)	‐
Asian	1.9% (*n* = 19/1024)	3.3% (*n* = 9/269)	‐	1.0% (*n* = 3/299)	2.3% (*n* = 7/305)	‐
Black	0.1% (*n* = 1/1024)	0% (*n* = 0/269)	‐	0.3% (*n* = 1/299)	0% (*n* = 0/305)	‐
Other	2.3% (*n* = 24/1024)	6.3% (*n* = 17/269)	‐	1.7% (*n* = 5/299)	0.6% (*n* = 2/305)	‐
Not recorded	91.0% (*n* = 932/1024)	90.0% (*n* = 242/269)	97.3% (*n* = 142/146)	91.6% (*n* = 274/299)	88.2% (*n* = 269/305)	100% (*n* = 5/5)
BMI, kg/m^2^	28.9 (24.3–33.4) [*s* = 88]	26.2 (22.2–31.5) [*s* = 29]	28.9 (26.6–38.0) [*s* = 23]	30.0 (28.0–33.5) [*s* = 31]	28.3 (28.0–28.6) [*s* = 4]	26.4 [*s* = 1]
Admission HbA1C, mmol/mol	77.5 (63.5–89.3) [*s* = 150]	80.0 (67.0–90.5) [*s* = 53]	78.5 (67.5–101.5) [*s* = 28]	75.0 (60.5–93.8) [*s* = 53]	75.5 (67.0–78.0) [*s* = 11]	62.0 (55.0–89.0) [*s* = 3]
Admission HbA1C, %	9.2 (8.0–10.3)	9.5 (8.3–10.4)	9.3 (8.3–11.4)	9.0 (7.7–10.7)	9.1 (8.3–9.3)	7.8 (7.2–10.3)
Duration of diabetes, years	10.0 (6.0–14.8) [*s* = 101]	10.0 (5.5–13.9) [*s* = 46]	9.9 (6.3–11.9) [*s* = 20]	10.0 (4.5–15.0) [*s* = 29]	13.3 (7.6–17.0) [*s* = 6]	NR [*s* = 0]
Duration of SGLT2i use, months	2.0 (0.5–10.8) [*s* = 88]	1.0 (0.25–10.0) [*s* = 39]	2.0 (0.3–9.9) [*n* = 11]	3.6 (1.0–12.0) [*s* = 32]	3.0 (0.25–87.2) [*s* = 5]	0.75 [*s* = 1]
SGLT2i agent						
Canagliflozin	20.1% (*n* = 206/1024)	4.1% (*n* = 11/269)	21.9% (*n* = 32/146)	44.1% (*n* = 132/299)	10.1% (*n* = 31/305)	0% (*n* = 0/5)
Dapagliflozin	23.5% (*n* = 241/1024)	34.2% (*n* = 92/269)	36.3% (*n* = 53/146)	17.1% (*n* = 51/299)	13.8% (*n* = 42/305)	60% (*n* = 3/5)
Empagliflozin	26.9% (*n* = 275/1024)	24.2% (*n* = 65/269)	39.7% (*n* = 58/146)	31.1% (*n* = 93/299)	18.7% (*n* = 57/305)	40% (*n* = 2/5)
Ertugliflozin	0.2% (*n* = 2/1024)	0% (*n* = 0/269)	0% (*n* = 0/146)	0.7% (*n* = 2/299)	0% (*n* = 0/305)	0% (*n* = 0/5)
Ipragliflozin	0.1% (*n* = 1/1024)	0.4% (*n* = 1/269)	0% (*n* = 0/146)	0% (*n* = 0/299)	0% (*n* = 0/305)	0% (*n* = 0/5)
Tofogliflozin	0.1% (*n* = 1/1024)	0.4% (*n* = 1/269)	0% (*n* = 0/146)	0% (*n* = 0/299)	0% (*n* = 0/305)	0% (*n* = 0/5)
Not recorded	29.1% (*n* = 298/1024)	36.7% (*n* = 99/269)	2.1% (*n* = 3/146)	7.0% (*n* = 21/299)	57.4% (*n* = 175/305)	0% (*n* = 0/5)
Biochemistry at presentation						
pH	7.1 (7.0–7.2) [*s* = 228]	7.1 (7.0–7.2) [*s* = 76]	7.1 (7.0–7.2) [*s* = 37]	7.2 (7.1–7.2) [*s* = 95]	7.2 (7.1–7.3) [*s* = 15]	7.2 (6.9–7.3) [*s* = 4]
Glucose, mmol/L	10.6 (8.8–14.0) [*s* = 238]	11.2 (9.3–13.7) [*s* = 78]	12.0 (9.3–15.7) [*s* = 35]	9.9 (8.2–12.6) [*s* = 104]	12.3 (9.4–16.2) [*s* = 16]	11.2 (7.2–19.9) [*s* = 4]
Glucose, mg/dL	190.8 (158.4–252.0)	201.6 (167.4–246.6)	216.0 (167.4–282.6)	178.2 (147.6–226.8)	221.4 (169.2–291.6)	201.6 (129.6–358.2)
Serum ketones, mmol/L	5.7 (4.4–8.1) [*s* = 151]	5.8 (4.0–9.1) [*s* = 45]	5.4 (4.8–6.5) [*s* = 18]	6.3 (4.5–8.4) [*s* = 72]	5.2 (4.6–6.5) [*s* = 14]	3.2 [*s* = 1]
Bicarbonate, mmol/L	8.8 (6.0–12.0) [*s* = 216]	8.1 (5.9–11.6) [*a* = 68]	7.8 (4.6–10.9) [*s* = 35]	9.0 (6.0–13.8) [*s* = 92]	10.0 (9.4–13.5) [*s* = 16]	5.5 (1.9–10.4) [*s* = 4]
Lactate, mmol/L	1.5 (1.0–2.3) [*s* = 134]	1.6 (1.1–2.5) [*s* = 50]	1.3 (0.9–2.1) [*s* = 29]	1.4 (1.1–2.2) [*s* = 47]	1.1 (0.7–1.5) [*s* = 3]	1.05 (0.8–1.1) [*s* = 3]
Sodium, mmol/L	137.0 (133.8–140.0) [*s* = 120]	137.8 (135.0–141.0) [*s* = 44]	136.0 (133.0–140.0) [*s* = 21]	136.0 (133.0–139.0) [*s* = 49]	142.5 (142.0–143.0) [*s* = 2]	135.0 (132.0–142.0) [*s* = 3]
Potassium, mmol/L	4.5 (3.9–5.1) [*s* = 128]	4.5 (3.8–5.0) [*s* = 48]	4.7 (4.1–5.4) [*s* = 22]	4.4 (4.0–5.1) [*s* = 50]	4.4 (4.3–5.3) [*s* = 3]	4.0 (3.7–5.2) [*s* = 4]
Urea, mmol/L	7.9 (4.6–11.3) [*s* = 71]	8.2 (4.6–11.4) [*s* = 31]	9.0 (4.5–18.0) [*s* = 12]	6.8 (3.8–9.6) [*s* = 25]	NR	6.7 (5.3–8.1) [*s* = 2]
Creatinine, μmol/L	94.1 (71.6–123.0) [*s* = 120]	90.6 (70.1–109.8) [*s* = 40]	94.0 (72.6–138.5) [*s* = 21]	106.1 (72.0–130.6) [*n* = 52]	74.0 (62.7–86.0) [*s* = 3]	84.9 (70.7–168.0) [*s* = 3]
Osmolality, mOsm/kg	304.0 (293.8–319) [*s* = 36]	308.4 (285.0–330.0) [*s* = 15]	294.0 (291.4–311.0) [*s* = 5]	305.0 (297.0–319.0) [*s* = 15]	NR	300.3 [*s* = 1]
Anion gap, mmol/L	24.0 (19.9–29.1) [*s* = 176]	25.0 (20.0–29.8) [*s* = 60]	24.8 (19.9–29.2) [*s* = 24]	23.0 (19.0–29.5) [*s* = 89]	22.1 (19.9–27.1) [*s* = 8]	28.5 (26.7–32.0) [*s* = 3]
Management and outcome						
Critical care admission, % (*n*)	21.1% (*n* = 216/1024)	35.7% (*n* = 96/269)	24.0% (*n* = 35/146)	17.7% (*n* = 53/299)	9.2% (*n* = 28/305)	80% (*n* = 4/5)
Episodes of Hypoglycaemia	0.3% (*n* = 3/1024)	0% (*n* = 0/269)	0% (*n* = 0/146)	0.3% (*n* = 1/299)	0.7% (*n* = 2/305)	0% (*n* = 0/5)
Episodes of Hypokalaemia	2.9% (*n* = 30/1024)	3.7% (*n* = 10/269)	1.4% [*n* = 2/146]	4.3% (*n* = 13/299)	0.7% (*n* = 2/305)	40% (*n* = 2/5)
Episodes of Hyperkalaemia	1.9% (*n* = 19/1024)	2.2% (*n* = 6/269)	3.4% (*n* = 5/146)	2.3% (*n* = 7/299)	0% (*n* = 0/305)	20% (*n* = 1/5)
DKA duration, hours	48.0 (23.8–60.5) [*s* = 106]	30.0 (23.3–61.5) [*s* = 32]	48.0 (30.0–81.0) [*s* = 17]	48.0 (19.8–48.0) [*s* = 47]	48.0 (19.7–72.0) [*s* = 7]	NR
Length of stay, days	7.5 (4.6–11.3) [*s* = 96]	10.1 (5.9–61.5) [*s* = 32]	9.0 (4.0–13.0) [*s* = 17]	5.0 (4.0–8.0) [*s* = 37]	9.4 (6.4–12.0) [*s* = 6]	9.8 (6.1–17.5) [*s* = 4]
Mortality, % (*n*)	0.7% (*n* = 7/1024)	0% (*n* = 0/269)	0% (*n* = 0/146)	1.3% (*n* = 4/299)	1.0% (*n* = 3/305) [*s* = 3]	0% (*n* = 0/5)

*Note*: [*s*]: number of studies that reported a value for the parameter. [*n*]: number of individuals that presented with DKA. Parameters are reported as (%, *n*) and median (IQR). For parameters with *n* = 3 or fewer samples, the data are reported as median (range) and as a single value for samples where *n* = 1. References are summarised in the Supplementary Material 2 [1–247].

Abbreviation: NR, not reported.

Admission biochemistry showed significant acidosis (median pH: 7.1), elevated serum ketones (5.7 mmol/L), and modestly raised blood glucose concentrations (median 10.6 mmol/L (190.8 mg/dL)), typical of SGLT2i‐associated DKA. Onset generally occurred after a median of 2 months of SGLT2i use. Regional trends indicated that Oceania had the highest admission glucose levels (12.3 mmol/L). The median duration of DKA and length of hospital stay were 48 h and 7.5 days, respectively, with regional variations. Mortality rates remained low, at 0.7%. Critical care admission was necessary in 21.1% of cases, with Asia and South America reporting the highest frequencies. Hypokalaemia and hypoglycaemia were rare during treatment.

### 3.2 DEKODE cohort data

Baseline cohort clinical characteristics: The baseline characteristics of the unmatched population are summarised in Supplementary Material 6. SGLT2i users and non‐SGLT2i users (controls) were matched in a 1:1 ratio, yielding an overall matched population of 534 individuals. In the subgroup analysis, SGLT2i users with a precipitating cause for DKA were matched 1:1 with those whose DKA was considered to be directly attributable to SGLT2i use, resulting in 81 individuals in each group. Additionally, SGLT2i users with a precipitating cause (*n* = 186) were matched 1:1 with SGLT2i non‐users. After matching for age, ethnicity, BMI, and CCI, all groups showed no differences in baseline characteristics (Table 2).

**TABLE 2:** Baseline characteristics of DKA cases from DEKODE database—matched for age, ethnicity, BMI and CCI.

Parameter	Overall [*n* = 534]	SGLT2i non‐users [*n* = 267]	SGLT2i users [*n* = 267]	*p*‐value	SGLT2i non‐users [*n* = 186]	SGLT2i (with precipitating cause) [*n* = 186]	*p*‐value	SGLT2i (with precipitating cause) [*n* = 81]	SGLT2i (related) [*n* = 81]	*p*‐value
Gender (Female), [%, *n*]	46.1% (*n* = 246)	45.7% (*n* = 122)	46.4% (*n* = 124)	0.931	37.1% (*n* = 69)	41.9% (*n* = 78)	0.396	49.4% (*n* = 40)	56.8% (*n* = 46)	0.431
Age (mean (SD)), [years]	61.5 (13.3)	61.9 (13.9)	61.1 (12.7)	0.511	62.1 (14.8)	61.7 (12.3)	0.769	59.8 (11.8)	59.7 (13.6)	0.961
Ethnicity (%)				0.981			0.712			0.520
Asian	9.6% (*n* = 51)	9.0% (*n* = 24)	10.1% (*n* = 27)		8.6% (*n* = 16)	11.8% (*n* = 22)		7.4% (*n* = 6)	6.2% (*n* = 5)	
Black	4.5% (*n* = 24)	4.1% (*n* = 11)	4.9% (*n* = 13)		5.4% (*n* = 10)	4.8% (*n* = 9)		1.2% (*n* = 1)	4.9% (*n* = 4)	
Unknown	5.8% (*n* = 31)	6.0% (*n* = 16)	5.6% (*n* = 15)		6.5% (*n* = 12)	5.4% (*n* = 10)		8.6% (*n* = 7)	6.2% (*n* = 5)	
Other	3.0% (*n* = 16)	3.0% (*n* = 8)	3.0% (*n* = 8)		2.2% (*n* = 4)	3.8% (*n* = 7)		0% (*n* = 0)	1.2% (*n* = 1)	
White	77.2% (*n* = 412)	77.9% (*n* = 208)	76.4% (*n* = 204)		77.4% (*n* = 144)	74.2% (*n* = 138)		82.7% (*n* = 67)	81.5% (*n* = 66)	
BMI (mean (SD)), [kg/m^2^]	29.9 (10.2)	30.2 (10.9)	29.6 (9.4)	0.518	30.0 (10.1)	29.8 (9.7)	0.861	29.4 (9.7)	29.3 (8.9)	0.971
CCI [mean (SD)]				0.605			0.977			0.683
<3 points	16.1% (*n* = 86)	16.5% (*n* = 44)	15.7% (*n* = 42)		14.5% (*n* = 27)	15.1% (*n* = 28)		17.3% (*n* = 14)	17.3% (*n* = 14)	
>3 points	27.2% (*n* = 145)	28.8% (*n* = 77)	25.5% (*n* = 68)		22.0% (*n* = 41)	22.6% (*n* = 42)		38.3% (*n* = 31)	32.1% (*n* = 26)	
Unknown	56.7% (*n* = 303)	54.7% (*n* = 146)	58.8% (*n* = 157)		63.4% (*n* = 118)	62.4% (*n* = 116)		44.4% (*n* = 36)	50.6% (*n* = 41)	

Abbreviations: BMI, body mass index; CCI, Charlson co‐morbidity index.

Intercurrent illness and suboptimal diabetes management were the most common DKA precipitants, and SGLT2i use, without any other identified precipitant, was the suspected cause of 30.3% of episodes (Supplementary Material 7). Biochemical profiles at admission showed that SGLT2i users had lower glucose, pH, bicarbonate, and potassium levels than non‐users (Table 3). Subgroup analysis revealed lower lactate and osmolality in cases where SGLT2i use was the sole precipitant. Management in SGLT2i users required a lower insulin infusion rate without differences in overall appropriateness or outcomes (Supplementary Material 8).

**TABLE 3:** Differences in admission biochemistry of DKA cases from DEKODE database between SGLT2i users and SGLT2i non‐users; non‐SGLT2i users and SGLT2i (with precipitating cause) and SGLT2i‐related DKA and SGLT2i (with precipitating cause—matched for age, gender, ethnicity, BMI, and precipitating cause).

Parameter	Overall [*n* = 534]	SGLT2i non‐users [*n* = 267]	SGLT2i users [*n* = 267]	*p*‐value	SGLT2i non‐users [*n* = 186]	SGLT2i (with precipitating cause) [*n* = 186]	*p*‐value	SGLT2i (with precipitating cause) [*n* = 81]	SGLT2i (related) [*n* = 81]	*p*‐value
Glucose [mmol/L] (mean (SD))	22.8 (10.7)	25.7 (10.4)	20.0 (10.2)	<0.001	26.4 (10.4)	21.6 (10.7)	<0.001	23.6 (12.0)	16.3 (8.1)	<0.001
Ketones [mmol/L] (mean (SD))	5.5 (1.5)	5.4 (1.6)	5.5 (1.5)	0.462	5.4 (1.5)	5.5 (1.5)	0.806	5.5 (1.6)	5.6 (1.4)	0.535
pH (mean (SD))	7.2 (0.2)	7.2 (0.2)	7.1 (0.2)	0.046	7.2 (0.1)	7.1 (0.2)	0.012	7.1 (0.1)	7.1 (0.2)	0.498
Bicarbonate [mmol/L] (mean (SD))	11.6 (5.3)	12.2 (4.9)	11.1 (5.6)	0.024	12.5 (5.2)	11.2 (5.5)	0.020	10.9 (5.3)	10.9 (5.8)	0.993
Lactate [mmol/L] (mean (SD))	3.1 (2.5)	3.5 (2.9)	2.7 (2.0)	<0.001	3.8 (3.3)	3.0 (2.3)	0.013	3.2 (2.9)	2.1 (1.0)	0.001
Sodium [mmol/L] (mean (SD))	134.7 (7.1)	133.7 (7.6)	135.6 (6.5)	0.002	134.1 (7.8)	135.1 (6.7)	0.172	135.1 (7.1)	136.9 (5.8)	0.089
Potassium [mmol/L] (mean (SD))	4.7 (1.0)	4.8 (1.0)	4.6 (1.1)	0.006	4.9 (1.0)	4.7 (1.1)	0.079	4.5 (1.1)	4.3 (0.8)	0.079
Urea [mmol/L] (mean (SD))	11.9 (9.2)	12.0 (8.8)	11.8 (9.6)	0.839	12.7 (8.5)	13.4 (10.6)	0.445	14.3 (12.1)	8.2 (4.7)	<0.001
Osmolality [mOsm/kg] (mean (SD))	303.9 (19.5)	305.0 (20.4)	302.9 (18.7)	0.217	306.8 (22.9)	305.0 (19.6)	0.406	308.1 (19.4)	298.0 (15.3)	<0.001

SGLT2i users experienced greater episodes of hypokalaemia (48.7% vs. 38.6%), and fewer episodes of hyperkalaemia (20.6% vs. 30.7%) compared to non‐users (Table 4). There was no difference in length of hospital stay between SGLT2i users and non‐users (10.5 vs. 13.4 days, *p* = 0.054); however, SGLT2i users required fewer ICU admissions (17.2% vs. 18.4%, *p* = 0.013). No significant differences in outcomes were observed between SGLT2i‐related DKA and DKA due to other causes (Table 4).

**TABLE 4:** Differences in complications and outcomes between SGLT2i users and SGLT2i non‐users; non‐SGLT2i users and SGLT2i (with precipitating cause) and SGLT2i‐related DKA and SGLT2i (with precipitating cause)—matched for age, gender, ethnicity, BMI, CCI, precipitating cause and admission biochemistry.

Parameter	Overall [*n* = 534]	SGLT2i non‐users [*n* = 267]	SGLT2i users [*n* = 267]	*p*‐value	SGLT2i non‐users [*n* = 186]	SGLT2i (with precipitating cause) [*n* = 186]	*p*‐value	SGLT2i (with precipitating cause) [*n* = 81]	SGLT2i (related) [*n* = 81]	*p*‐value
Hypoglycaemia (%, *n*)	13.3% (*n* = 71)	12.7% (*n* = 34)	13.9% (*n* = 37)	0.799	11.3% (*n* = 23)	14.0% (*n* = 26)	0.532	9.9% (*n* = 8)	13.6% (*n* = 11)	0.625
Hypokalaemia (%, *n*)	43.6% (*n* = 233)	38.6% (*n* = 103)	48.7% (*n* = 130)	0.023	32.8% (*n* = 61)	45.7% (*n* = 85)	0.015	54.3% (*n* = 44)	55.6% (*n* = 45)	1.00
Hyperkalaemia (%, *n*)	25.7% (*n* = 137)	30.7% (*n* = 82)	20.6% (*n* = 55)	0.010	25.3% (*n* = 47)	25.3% (*n* = 47)	1.00	21.0% (*n* = 17)	9.9% (*n* = 8)	0.082
DKA duration [hours], (mean (SD))	21.2 (19.1)	21.0 (21.5)	21.4 (16.4)	0.822	21.5 (24.7)	21.4 (16.9)	0.945	21.1 (16.2)	21.4 (15.2)	0.878
ITU admission, (%, *n*)	17.8% (*n* = 95)	18.4% (*n* = 49)	17.2% (*n* = 46)	0.013	21.0% (*n* = 39)	18.3% (*n* = 34)	0.426	21.0% (*n* = 17)	14.8% (*n* = 12)	0.336
Length of Stay [days], (mean (SD))	12.0 (17.7)	13.4 (19.9)	10.5 (15.2)	0.054	12.2 (18.3)	11.3 (13.7)	0.619	13.2 (17.7)	8.5 (18.2)	0.097
Mortality (%, *n*)	7.1% (*n* = 38)	6.7% (*n* = 18)	7.5% (*n* = 20)	0.866	6.5% (*n* = 12)	9.1% (*n* = 17)	0.439	8.6% (*n* = 7)	3.7% (*n* = 3)	0.327

## 4 DISCUSSION

Within the DEKODE cohort, we demonstrate that DKA in individuals with type 2 diabetes treated with SGLT2 inhibitors is characterised by a distinct biochemical profile with notably lower blood glucose levels and more pronounced acidosis compared to SGLT2i non‐users. However, despite these biochemical differences, clinical outcomes—including length of stay, complications, and mortality—were broadly comparable. The atypical presentation of euglycaemic DKA may facilitate earlier diagnosis, as clinicians maintain a high index of suspicion and potentially trigger more rapid initiation of treatment, which may mitigate progression to severe complications.^2^ In addition, SGLT2i promote osmotic diuresis and natriuresis by inhibiting renal glucose reabsorption, resulting in increased urinary glucose and sodium excretion.^26^ This altered fluid balance may predispose patients to volume depletion and electrolyte disturbances, such as hypokalaemia or hyperkalaemia, which can independently contribute to clinical severity despite lower glycaemia. The hormonal milieu in SGLT2i‐treated individuals is altered, characterised by a reduced insulin‐to‐glucagon ratio, favouring lipolysis and ketone body production.^27^ This metabolic shift may exacerbate ketogenesis and acidosis even in the absence of severe hyperglycaemia.

Despite SGLT2i users presenting with worse acidosis, ketone levels at presentation were similar between SGLT2i users and non‐users. This has been previously reported in smaller studies and may suggest that healthcare professionals appropriately identify DKA in SGLT2i users before the development of more severe ketonaemia.^20^ Individuals with SGLT2i‐related DKA were less likely to be hypovolaemic, as inferred by lower urea and osmolality values. This contrasts with Almazrouei et al., who reported a higher hypovolaemia burden in SGLT2i users.^19^ SGLT2i, through their promotion of glycosuria, are mechanistically associated with osmotic diuresis and are thus expected to predispose to dehydration. Our findings may be attributed to several factors, such as a slower or more insidious onset of DKA, which may allow physiological compensatory mechanisms to preserve intravascular volume.

Within the DEKODE cohort, individuals with SGLT2i‐associated DKA presented with lower serum potassium levels, had a greater proportion of hypokalaemia events, and fewer hyperkalaemia events during the DKA episode. Several interrelated mechanisms can explain this paradox. First, SGLT2 inhibitors induce glycosuria‐driven osmotic diuresis, leading to increased urinary potassium losses. Second, the associated natriuresis and volume contraction activate the renin–angiotensin–aldosterone system, enhancing renal potassium excretion. Following initiation of insulin therapy, the intracellular redistribution of potassium can further precipitate hypokalaemia. Collectively, these mechanisms highlight the need for close monitoring and proactive correction of potassium levels during the management of SGLT2i‐associated DKA.^28^

Importantly, intercurrent illness and suboptimal diabetes management were identified as precipitating factors in over two‐thirds of cases, highlighting key opportunities for risk mitigation and prevention. Other studies report prolonged fasting, surgical intervention, and infection as common precipitants.^29^, ^30^, ^31^ This highlights the need for targeted education for healthcare providers and people with type 2 diabetes to raise awareness and prevent SGLT2i‐related DKA. Individuals who may be at higher risk of ketoacidosis include those with a low beta‐cell function reserve (e.g., type 2 diabetes patients with low C‐peptide, latent autoimmune diabetes in adults (LADA) or patients with a history of pancreatitis); patients on very low carbohydrate and ketogenic diets, patients on calorie‐restricted diets, patients with conditions that lead to restricted food intake or severe dehydration; or those undergoing surgery. SGLT2 inhibitors should be used with caution in these patient groups.^29^ International Consensus guidelines for SGLT2i‐related DKA in T1D have developed mnemonics such as STICH^32^ and STOP DKA^33^ to streamline protocols and empower individuals to act efficiently if experiencing symptoms of DKA. A similar approach, alongside comprehensive sick day rule guidance, may be needed for people with type 2 diabetes.^34^

Despite the propensity of SGLT2is to precipitate DKA, clinical outcomes such as ICU admission rates and hospital length of stay were not worse. We observed a significantly lower proportion of ICU admissions among SGLT2i users (*p* = 0.013), which could be attributed to earlier recognition of euglycaemic DKA, variations in clinical presentation, or more timely application of treatment guidelines; however, this observation is likely limited by the sample size and warrants further investigation. Although the reduction in hospital stay did not reach statistical significance (*p* = 0.054), the near‐threshold value suggests that further studies with larger cohorts are needed to clarify these trends.

The meta‐summary, reinforced by data from the DEKODE cohort, provides further comprehensive analysis. It is the first study to differentiate between DKA cases that are suspected to be attributed to SGLT2i use and those with additional precipitating factors and directly comparing them to SGLT2i non‐users. The cases in the meta‐summary had a longer DKA duration compared to the DEKODE cohort. DKA cases within the DEKODE cohort were managed according to guidelines similar to the newly proposed international consensus, which may explain the improved outcomes in those scenarios. However, patients in the DEKODE cohort had longer hospital stays compared to those in the meta‐summary. Since there were no differences in presentation, DKA duration, or baseline comorbidities (as indicated by similar CCI scores), conditions related to precipitating factors of DKA, rehabilitation, or social factors may have contributed to the longer stays in the real world.

Additionally, the meta‐summary suggested that most patients were in the euglycaemic range, a finding not seen within the DEKODE cohort, where patients exhibited higher admission glucose. This may be due to selective reporting, as euglycemic DKA is more frequently documented through individual case reports, whereas typical hyperglycaemic DKA is less often reported in this manner. This may also result from individual variations in hepatic glycogen stores, glucose production, and renal glucose excretion.^21^, ^29^ It is important to emphasise this phenomenon, as SGLT2i‐associated DKA may be overlooked if it does not conform to the euglycemic DKA definition. Nevertheless, individuals with DKA suspected to be directly related to SGLT2i had lower glucose levels than those with DKA triggered by an additional cause and SGLT2i non‐users.

The strengths of our study include its comprehensive meta‐summary, multi‐centre design, large sample size, and the inclusion of biochemical and outcome data for real‐world clinical applications. The meta‐summary represents the most extensive analysis of SGLT2 inhibitor‐associated DKA in people with type 2 diabetes to date, highlighting regional differences in DKA precipitants and outcomes. The DEKODE collaboration operates a standardised data collection system, and participating centres confirmed that all DKA admissions were included. We could not delineate the episodes of DKA by individual classes of SGLT2i due to a lack of data regarding the specific type of SGLT2i. However, a study across three US healthcare databases revealed no differential increase in DKA between canagliflozin, empagliflozin, and dapagliflozin.^35^

The absence of data on admission HbA1C, duration of SGLT2i therapy prior to the DKA episode, duration of T2D, number of previous DKA episodes, and presenting symptoms in real‐world data may restrict further interpretation of our findings. In addition, we were unable to perform propensity scoring and matching based upon time‐varying confounders, including duration of SGLT2i use and HbA1C trends. An important limitation of our study is the lack of detailed data on SGLT2 inhibitor dosage and treatment duration, which prevented assessment of potential dose‐related DKA risk. Given that pharmacodynamic effects may vary with dose, including impacts on ketogenesis, insulin sensitivity, and volume status, this represents a key area for future investigation. We acknowledge that establishing direct causality of SGL2i‐related DKA is not possible within this retrospective study, alongside stratification of outcomes by precipitant, particularly due to small sample sizes that may have limited statistical power, reliance on patient‐reported histories, and treating physician documentation. As such, statistical findings should be interpreted with caution. Reported p‐values reflect nominal statistical significance only, and results should be viewed as exploratory rather than confirmatory.

## 5 CONCLUSION

Despite more severe metabolic acidosis in SGLT2i users, outcomes such as ICU admission, length of stay, and mortality were comparable to those of non‐users. Most DKA episodes in SGLT2i users were triggered by factors unrelated to the medication itself. These findings highlight the need for improved clinical awareness, risk stratification, and targeted education to better prevent and manage SGLT2i‐related DKA, thereby preserving the cardio‐renal benefits of these medications.

## AUTHOR CONTRIBUTIONS

AS was involved in data collection, analysis, and manuscript writing. SAB, LR, NP, AM, and AA were involved in data collection. RT performed and supervised the statistical analyses. PN, KD, and GEU provided critical input on the study design, analysis, and manuscript writing. PK conceptualised and supervised the study's design, data collection, analysis, and manuscript writing. The members of the DEKODE team were involved in data collection and implementation of guidelines. All of them qualify for shared authorship of this paper. The team includes Anjitha Anilkumar, Anmol Bagga, Ankita Gupta, Canay Onder, Carina Synn Cuen Pan, Catherine Cooper, Dineshwaran Rajendran, Dolu Falowo, Eka Melson, Emily Warmington, Francesca Pang, Jason Cheung, Kaushal Maru, Lucy Bomphrey, Manbir Duggal, Meghna Hebbar, Megan Owen, Muhammad Ali Karamat, My Chi Pham, Pranav Viswanath Iyer, Ragavendran Govindaraj Sureshkumar, Saima Kauser Malik, Sanjay Saraf, Senthil Kumar, Shamanth Soghal, Shivam Choudhary, and Wai Nga Alice Yip.

## FUNDING INFORMATION

This research was supported by the National Institute for Health and Care Research (NIHR) Advanced Clinician Scientist Fellowship awarded to author PK, ABCD and Sanofi DKA Collaborative Working Project Grant and Midlands Patient Safety Research Collaboration (PSRC). The views expressed are those of the author(s) and not necessarily those of the NIHR or the Department of Health and Social Care.

## CONFLICT OF INTEREST STATEMENT

The authors declare no conflicts of interest.

## PEER REVIEW

The peer review history for this article is available at https://www.webofscience.com/api/gateway/wos/peer-review/10.1111/dom.70098.

## Supporting information


**Data S1:** Supporting information

## Data Availability

The data that supports the findings of this study are available in the supplementary material of this article and are available from the corresponding author upon reasonable request.
