# Supplementary material for: Clinical characteristics and outcomes of diabetes‐related ketoacidosis (DKA) in sodium‐glucose co‐transporter‐2 inhibitor (SGLT2i) users with type 2 diabetes
Source: Diabetes Obes Metab. 2025 Sep 8;27(12):6999–7009. doi: 10.1111/dom.70098 (PMC12587257; doi:10.1111/dom.70098)
Supplement: Supplementary file 1 — Data S1: Supporting information [file DOM-27-6999-s001.docx]

**Supplementary Material**

**Supplementary Material 1**: Search strategies used for the systematic review.

| Database | Search Strategy |
| --- | --- |
| PubMed/COCHRANE | 1 Diabetic ketoacidosis [mh]  2 Diabetes ketoacidosis [tw] OR DKA [tw] OR euglycaemic DKA [tw] OR ketoacidosis [tw] OR euglycaemic ketoacidosis [tw]  3 1 OR 2  4 SGLT [tw]  5 SGLT2 [tw]  6 Sodium-Glucose Transport Proteins [mh]  7 “Sodium-Glucose Transport Protein*” OR "Sodium-glucose transporter" OR “Sodium-glucose co-transporter” OR “Sodium glucose cotransporter”  8 gliflozins[mh]  9 gliflozins[tw]  10 bexagliflozin[mh]  11 bexagliflozin[tw]  12canagliflozin[mh] 13 canagliflozin[tw]  14 dapagliflozin[mh] 15 dapagliflozin[tw]  16 empagliflozin[mh] 17 empagliflozin[tw]  18 ertugliflozin[mh]  19 ertugliflozin[tw]  20 henagliflozin[mh]  21 henagliflozin[tw]  22ipragliflozin[mh] 23 ipragliflozin[tw]  24 licogliflozin[mh]  25 licogliflozin[tw]  26 luseogliflozin[mh]  27 luseogliflozin[tw]  28 remogliflozin[mh] 29 remogliflozin[tw]  30 sergliflozin[mh] 31 sergliflozin[tw]  32 sotagliflozin[mh]  33 sotagliflozin[tw]  34 tofogliflozin[mh] 35 tofogliflozin[tw]  36 gliflozin[mh]  37 gliflozin[tw]  38 OR/4-37  39 3 AND 38 |
| EMBASE/ MEDLINE (via OVID)/ SCOPUS/ Web of Science | 1 Exp diabetic ketoacidosis/  2 DKA or diabetes ketoacidosis or euglycaemic DKA or euglycaemic ketoacidosis or ketoacidosis.mp.  3 1 or 2  4 exp sodium glucose cotransporter 2/ or exp sodium glucose cotransporter 2 inhibitor/  5 sglt.mp 6 sglt-2.mp. 7 sglt2.mp.  8 Sodium-Glucose Transport Protein.mp. 9 Sodium-glucose transporter.mp. 10 Sodium-glucose co-transporter.mp. 11 Sodium glucose cotransporter.mp.  12 gliflozins.mp. or exp gliflozins/  13 bexagliflozin.mp. or exp bexagliflozin/  14 canagliflozin.mp. or exp canagliflozin/  15 dapagliflozin.mp. or exp dapagliflozin/  16 empagliflozin.mp. or exp empagliflozin/  17 ertugliflozin.mp. or exp ertugliflozin/  18 henagliflozin.mp. or exp henagliflozin/  19 ipragliflozin.mp. or exp ipragliflozin/  20 licogliflozin.mp. or exp licogliflozin/  21 luseogliflozin.mp. or exp luseogliflozin/  22 remogliflozin.mp. or exp remogliflozin etabonate/  23 sergliflozin.mp. or exp sergliflozin etabonate/  24 sotagliflozin.mp. or exp sotagliflozin/  25 tofogliflozin.mp. or exp tofogliflozin/  26 gliflozin.mp. or exp gliflozin/  27 4 or 5 or 6 or 7 or 8 or 9 or 10 or 11 or 12 or 13 or 14 or 15 or 16 or 17 or 18 or 19 or 20 or 21 or 22 or 23 or 24 or 25 or 26  28 3 and 27 |

**Supplementary Material 2:** PRISMA Flow Diagram of Study Selection for the systematic review and Meta-Summary


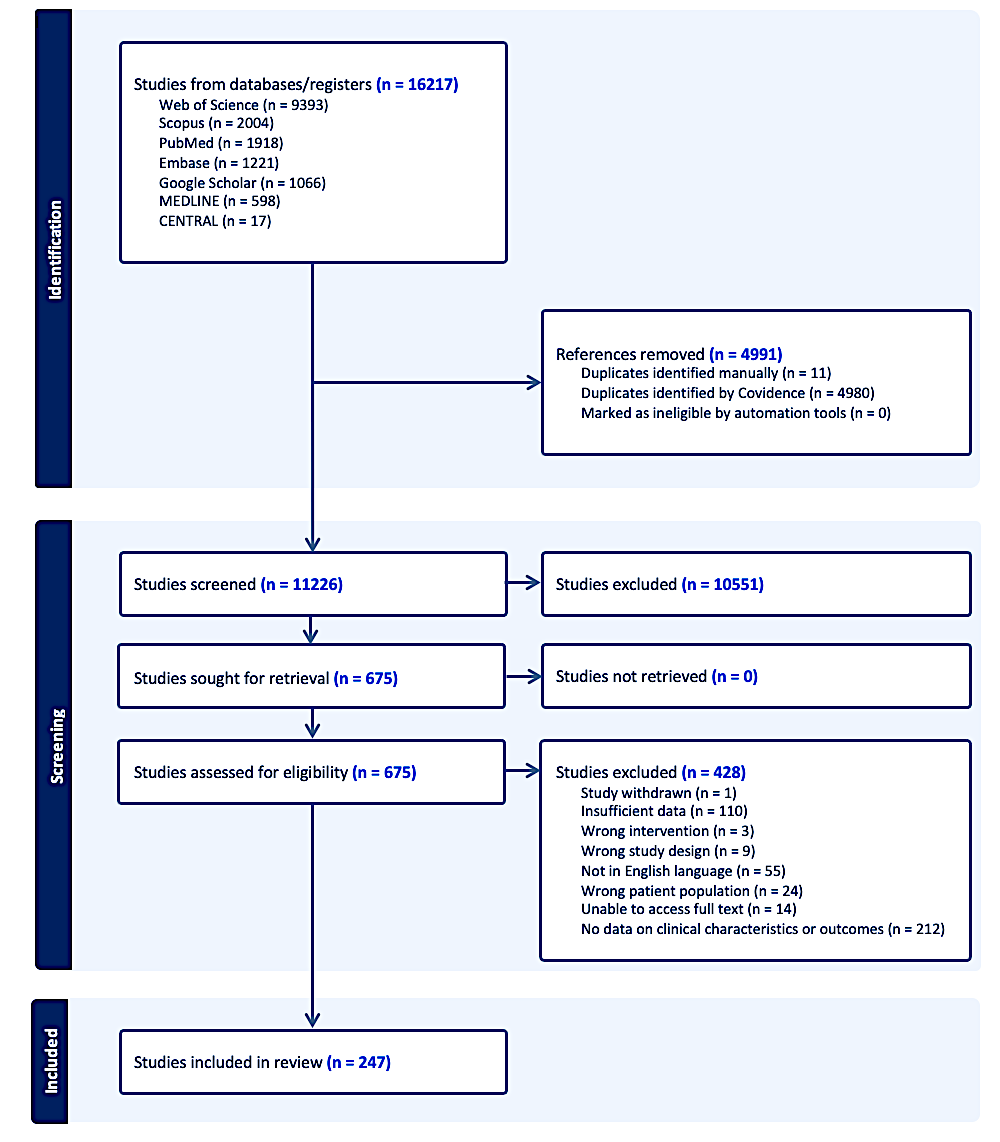


**Supplementary Material 3:** A map to illustrate cases of diabetes ketoacidosis (DKA) in individuals with Type 2 Diabetes on SGLT2-inhibitor therapy.

**
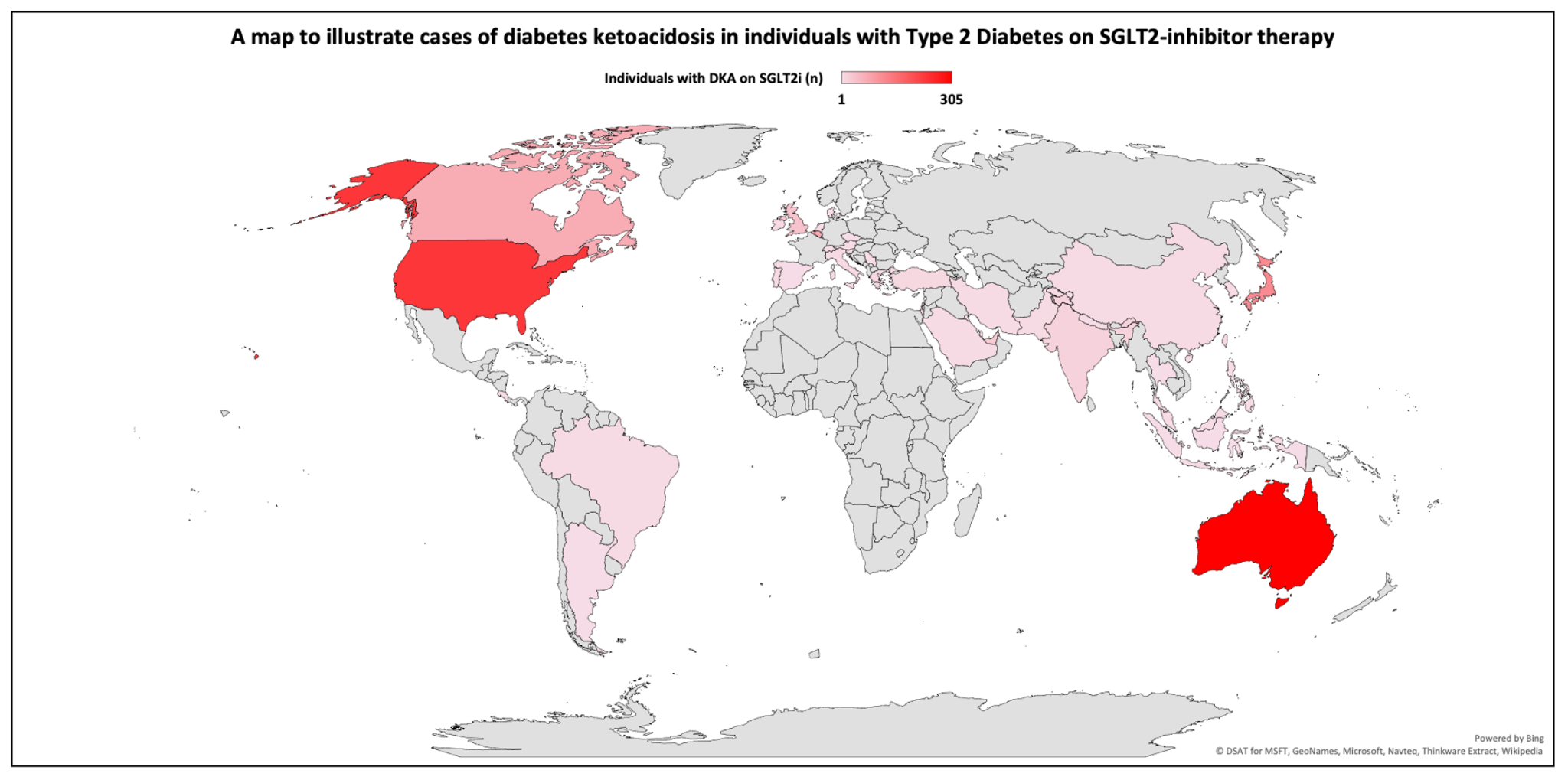
**

**Supplementary Material 4: References for meta-summary** [1-247].

[1] Kinoshita M, Azuma K, Yanagawa T. A case of sodium-glucose cotransporter-2 inhibitor-associated euglycemic diabetic ketoacidosis complicated by concurrent subacute thyroiditis. *J Clin Transl Endocrinol Case Rep*. 2022;25((Kinoshita, Azuma, Yanagawa) Department of Internal Medicine, Nerima General Hospital, Ashahigaoka, Nerima-ku, Tokyo 1-24-1, Japan(Kinoshita) 35th Medical Group, Misawa Air Base, Building 99, Unit 5024, Misawa Air Base, PO, AP 96319, Japan(Yanagawa) Insti):100124. doi:https://dx.doi.org/10.1016/j.jecr.2022.100124

[2] Dai Z, Nishihata Y, Kawamatsu N, Komatsu I, Mizuno A, Shimizu M, et al. Cardiac arrest from acute myocardial infarction complicated with sodium-glucose cotransporter 2 inhibitor-associated ketoacidosis. *J Cardiol Cases*. 2017;15(2):56-60. doi:10.1016/j.jccase.2016.10.006

[3] Tsutsui E, Hoshina Y, Homma H. Sodium-Glucose Cotransporter-2 Inhibitor-Induced Euglycemic Diabetic Ketoacidosis Followed by Excessively Low Carbohydrate Diet. *Cureus*. 2021;13(7):e16085-. doi:10.7759/cureus.16085

[4] Chou YM, Seak CJ, Goh ZNL, Seak JC, Seak CK, Lin CC. Euglycemic diabetic ketoacidosis caused by dapagliflozin: A case report. *Medicine (Baltimore)*. 2018;97(25):e11056-. doi:10.1097/MD.0000000000011056

[5] Khan A, Mushtaq K, Khakwani M, Khakwani MSK, Mushtaq R, Robison R, et al. Metabolic Acidosis and its Predisposing Factor: Euglycemic Ketoacidosis Caused by Empagliflozin and Low-Carbohydrate Ketogenic Diet in Type 2 Diabetes Mellitus. Case Report. *SN Compr Clin Med*. 2020;2(8):1243-1247. doi:https://dx.doi.org/10.1007/s42399-020-00367-0

[6] Han J, Xie S, Jiao H. Sodium-glucose cotransporter-2 inhibitor-induced euglycemic diabetic ketoacidosis: A case report. *J Clin Pharm Ther*. 2022;47(9):1482-1485. doi:10.1111/jcpt.13737

[7] Yeoh HL, Lee M, Pan WJ, Ong HY. Case of sodium-glucose cotransporter-2 inhibitor-associated euglycaemic diabetic ketoacidosis. *BMJ Case Rep*. 2021;14(8). doi:10.1136/bcr-2020-235953

[8] Almazrouei R, Afandi B, AlKindi F, Govender R, Al-Shamsi S. Clinical Characteristics and Outcomes of Diabetic Ketoacidosis in Patients With Type 2 Diabetes using SGLT2 Inhibitors. *Clin Med Insights Endocrinol Diabetes*. 2023;16:11795514231153716-. doi:10.1177/11795514231153717

[9] M. Abbas S, Jamal A. bagi F, Abdalkarim A, Sabir Balla N, Abdelrahman A, Y Fadul K. Case Report: Chest Pain as an Uncommon Presentation for Euglycemic Diabetic Ketoacidosis in a Female Patient Using SGLT2 inhibitor. *F1000Res*. 2023;12:1157.

[10] Miwa M, Nakajima M, Kaszynski RH, Goto H. Prolonged euglycemic diabetic ketoacidosis triggered by a single dose of sodium-glucose cotransporter 2 inhibitor. *BMJ Case Rep*. 2020;13(10). doi:10.1136/bcr-2020-235969

[11] Yii ESS, Azli AW, Sitaram PN. Sodium-glucose cotransporter 2 inhibitor-induced euglycemic diabetic ketoacidosis in a patient with coronavirus disease 2019: a case report. *J Med Case Rep*. 2022;16(1):17. doi:10.1186/s13256-021-03232-3

[12] Alkatheeri A, Alseddeeqi E. Euglycemic diabetic ketoacidosis induced by sodium-glucose cotransporter 2 inhibitor in the setting of prolonged fasting: a case report. *J Med Case Rep*. 2022;16(1):138. doi:10.1186/s13256-022-03347-1

[13] Wang Q, Wu K, Luo X, Dong X, Liu W, Tang Z, et al. Dapagliflozin-Associated Euglycemic Diabetic Ketoacidosis Presenting With Severe Abdominal Pain Mimicking Acute Peritonitis. *Cureus*. 2022;14(2):e22229-. doi:10.7759/cureus.22229

[14] Luo X, Ji R, Lu W, Zhu H, Li L, Hu J. Dapagliflozin-Associated Euglycemic Diabetic Ketoacidosis in a Patient Who Underwent Surgery for Pancreatic Carcinoma: A Case Report. *Front Surg*. 2022;9:769041. doi:10.3389/fsurg.2022.769041

[15] Qiang W, Yang F, Liu L, Dong R, Guo H. SGLT-2 inhibitors and high-dose acarbose as potential high-risk combi-nations for ketosis and ketoacidosis in Asian patients with T2DM: A case series. *J Clin Images Med Case Rep*. 2024;5(3):2950.

[16] Vadi S, Agarwal M. Canagliflozin-associated Diabetic Ketoacidosis with Lower-than-anticipated Glucose Levels. *Indian J Crit Care Med*. 2017;21(11):793-795. doi:10.4103/ijccm.IJCCM_328_17

[17] Singh DG. DAPAGLIFLOZIN ASSOCIATED EUGLYCEMIC DIABETIC KETOACIDOSIS: A CASE REPORT. Published online 2022.

[18] Shah M, Pathrose E, Bhagwat NM, Chandy D. “The Bitter Truth of Sugar”-Euglycemic Diabetic Ketoacidosis due to Sodium-glucose Cotransporter-2 Inhibitors: A Case Series. *Indian J Crit Care Med*. 2022;26(1):123-126. doi:10.5005/jp-journals-10071-24076

[19] Kela MM. Dapagliflozin Causing Euglycemic Diabetic Ketoacidosis. *Journal of Research & Innovation in Anesthesia*. 2023;8(1):11-12.

[20] Ghosh A, Gupta R, Misra A. Ketonuria/ketonemia associated with the use of sodium-glucose cotransporter 2 (SGLT-2) inhibitors in type 2 diabetes: A report of three cases from New Delhi, India. *J Diabetes*. 2016;8(5):738-739. doi:10.1111/1753-0407.12411

[21] Kuchay MS, Mishra SK, Mehta Y. Empagliflozin induced euglycemic diabetic ketoacidosis in a patient undergoing coronary artery bypass graft despite discontinuation of the drug 48 hours prior to the surgery. *Diabetes Metab Syndr*. 2021;15(3):909-911. doi:10.1016/j.dsx.2021.04.016

[22] Vadi S, Lad V, Kapoor D. Perioperative Implication of Sodium-glucose Cotransporter-2 Inhibitor in a Patient Following Major Surgery. *Indian J Crit Care Med*. 2021;25(8):958-959. doi:10.5005/jp-journals-10071-23929

[23] Kamath SD, Kumar U, Shrivastava V. Sodium-Glucose Cotransporter 2 Inhibitor-Induced Euglycemic Diabetic Ketoacidosis: The Other Side of the Coin! *Cureus*. 2024;16(4):e58341-. doi:10.7759/cureus.58341

[24] M AG. A case of euglycemic diabetic keto acidosis. *Indian J Endocrinol Metab*. 2019;23(4):500-501. doi:https://dx.doi.org/10.4103/ijem.ijem_302_19

[25] Wibawa K, Kuhuwael F V, Putra CRJ, Widiastuti SU, Suciadi LP. Euglycemic diabetic ketoacidosis associated with empagliflozin in patients hospitalized with acute pulmonary embolism. *Clinical Diabetology*. 2021;10(2):204-208. doi:https://dx.doi.org/10.5603/DK.a2021.0010

[26] Mohamad Hosein Zade Davatgari R, Soti Khiabani M, Shahmirzalou P, Habibi M. Euglycemic Diabetic Ketoacidosis in a Type 2 Diabetic Patient Treated with Empagliflozin: Case Report. *Journal of Kerman University of Medical Sciences*. 2023;30(5):296-299.

[27] Shojaei L, Majidzadeh K. Empagliflozin induced diabetes ketoacidosis: a review of literature. Published online 2022.

[28] Abu-Amer N, Dinour D, Mini S, Beckerman P. An Unusual Case of Metabolic Acidosis: Clinical Case Education. *Isr Med Assoc J*. 2019;21(11):766-768.

[29] Fukuda M, Nabeta M, Muta T, Fukami K, Takasu O. Euglycemic diabetic ketoacidosis caused by canagliflozin: a case report. *Int J Emerg Med*. 2020;13(1):2. doi:10.1186/s12245-020-0261-8

[30] Seki H, Ideno S, Shiga T, Watanabe H, Ono M, Motoyasu A, et al. Sodium-glucose cotransporter 2 inhibitor-associated perioperative ketoacidosis: a systematic review of case reports. *J Anesth*. 2023;37(3):465-473. doi:10.1007/s00540-023-03174-8

[31] Miyauchi M, Toyoda M, Fukagawa M. Atypical Ketoacidosis and Protracted Hyperglycosuria after Treatment with Ipragliflozin, an SGLT2 Inhibitor. *Intern Med*. 2017;56(13):1673-1678. doi:10.2169/internalmedicine.56.7945

[32] Hashi R, Fujiki N, Yagi T. Tubular Injury Causing Protracted Glycosuria Following Withdrawal of a Sodium-Glucose Cotransporter 2 (SGLT2) Inhibitor: A Possible Role in the Development of Protracted Hypoglycemia and Ketoacidosis. *Tohoku J Exp Med*. 2021;255(4):291-296. doi:10.1620/tjem.255.291

[33] Kawahara J, Kaku B, Yagi K, Kitagawa N, Yokoyama M, Wakabayashi Y, et al. Life-threatening coronary vasospasm in patients with type 2 diabetes with SGLT2 inhibitor-induced euglycemic ketoacidosis: a report of two consecutive cases. *Diabetol Int*. 2024;15(1):135-140. doi:10.1007/s13340-023-00664-8

[34] Inoue A, Katayama A, Sue M, Hasegawa M, Maeda M, Matoba M, et al. A case of type 2 diabetes mellitus leading to euglycemic diabetic ketoacidosis in 3 days after starting sodium-glucose cotransporter 2 inhibitor while on a very-low-carbohydrate diet. *Authorea Preprints*. Published online 2022.

[35] Ito T, Sugasawa G, Suzuki F, Sunada M, Iwamuro K, Nakano T, et al. Insulin and glucose infusion could prevent euglycemic diabetic ketoacidosis associated with sodium-glucose cotransporter 2 inhibitors. *Indian J Thorac Cardiovasc Surg*. 2022;38(1):87-91. doi:10.1007/s12055-021-01227-9

[36] Kitahara C, Morita S, Kishimoto S, Matsuno S, Uraki S, Takeshima K, et al. Early detection of euglycemic ketoacidosis during thoracic surgery associated with empagliflozin in a patient with type 2 diabetes: A case report. *J Diabetes Investig*. 2021;12(4):664-667. doi:10.1111/jdi.13365

[37] Goto S, Ishikawa JY, Idei M, Iwabuchi M, Namekawa M, Nomura T. Life-Threatening Complications Related to Delayed Diagnosis of Euglycemic Diabetic Ketoacidosis Associated with Sodium-Glucose Cotransporter-2 Inhibitors: A Report of 2 Cases. *Am J Case Rep*. 2021;22:e929773-. doi:10.12659/AJCR.929773

[38] Hitsuwari T, Tsurutani Y, Yamane T, Sunouchi T, Horikoshi H, Hirose R, et al. Two Cases of Thyrotoxicosis and Euglycemic Diabetic Ketoacidosis Under Sodium-glucose Transport Protein 2 Inhibitor Treatment. *Intern Med*. 2022;61(20):3069-3075. doi:10.2169/internalmedicine.8830-21

[39] Taniguchi H, Terayama T, Seno S, Kiriu N, Kato H, Sekine Y, et al. Delayed diagnosis of severe diabetic ketoacidosis associated with a sodium-glucose cotransporter 2 inhibitor: a case report. *Oxf Med Case Reports*. 2023;2023(7):omad074-. doi:10.1093/omcr/omad074

[40] Yi WJ, Kim SK, Youn SU, Kang N, Lee MW, Park SO. Euglycemic Diabetic Ketoacidosis When Reducing Insulin Dosage in Patients Taking Sodium Glucose Cotransporter 2 Inhibitor. *EWHA MEDICAL JOURNAL*. 2017;40(1):55-58. doi:10.12771/emj.2017.40.1.55

[41] Yeo SM, Park H, Paek JH, Park WY, Han S, Park SB, et al. Ketoacidosis with euglycemia in a patient with type 2 diabetes mellitus taking dapagliflozin: A case report. *Medicine (Baltimore)*. 2019;98(3):e14150-. doi:10.1097/MD.0000000000014150

[42] Bitar ZI, Maadarani OS, Alabdali F, Teama A, Elsawah W, Mohsen MJ, et al. Sodium-glucose cotransporter-2 inhibitors induced euglycemic diabetic ketoacidosis: Two case reports and a review of the literature. *Clin Case Rep*. 2022;10(2):e05440-. doi:10.1002/ccr3.5440

[43] Maadarani O, Bitar Z, Alhamdan R. Dapagliflozin-induced severe ketoacidosis requiring hemodialysis. *Clin Med Rev Case Rep*. 2016;3:150.

[44] S WAS, Sukor N, Y ASM, Ismail I, Kamaruddin NA. Case Report: High-Calorie Glucose Infusion and Tight Glycemic Control in Ameliorating Refractory Acidosis of Empagliflozin-Induced Euglycemic Diabetic Ketoacidosis. *Front Endocrinol (Lausanne)*. 2022;13:867647. doi:10.3389/fendo.2022.867647

[45] Pathak BD, Dhakal B, Bhattarai AM, Regmi BU, Mandal SK, Panta PR, et al. Euglycemic diabetic ketoacidosis in a patient with acute stroke taking sodium glucose co-transporter 2 inhibitor. *Annals of Medicine and Surgery*. 2022;79((Pathak, Dhakal, Bhattarai, Regmi, Mandal, Panta, Simkhada) Nepalese Army Institute of Health Sciences, College of Medicine, Kathmandu, Nepal(Khadka) Department of Internal Medicine, Nepalese Army Institute of Health Sciences, College of Medicine, Kathman):104118. doi:https://dx.doi.org/10.1016/j.amsu.2022.104118

[46] Mahfooz RS, Khan MK, H AH, Khedr A. SGLT-2 Inhibitor-Associated Euglycemic Diabetic Ketoacidosis: A Case Report and a Literature Review. *Cureus*. 2022;14(6):e26267-. doi:10.7759/cureus.26267

[47] Rashid O, Farooq S, Kiran Z, Islam N. Euglycaemic diabetic ketoacidosis in a patient with type 2 diabetes started on empagliflozin. *BMJ Case Rep*. 2016;2016. doi:10.1136/bcr-2016-215340

[48] Sarno MJF, Hernandez DPF, Matulac MO. “Normal but Catastrophic” Euglycemic Diabetic Ketoacidosis Precipitated by Sodium-Glucose Cotransporter-2 Inhibitor Use: A Case Report. *Cureus*. 2023;15(11):e49236-. doi:10.7759/cureus.49236

[49] S EEM, ElRishi MA. Severe euglycemic diabetic ketoacidosis secondary to sodium-glucose co-transporter 2 inhibitor: case report and literature review. *Ann Med Surg (Lond)*. 2023;85(5):2097-2101. doi:10.1097/MS9.0000000000000479

[50] Razok A, Ata F, Ahmed SMI, DHSH AM. Sodium-glucose co-transporter 2 inhibitors induced euglycemic diabetic ketoacidosis within four days of initiation. *World J Diabetes*. 2022;13(3):272-274. doi:10.4239/wjd.v13.i3.272

[51] Ata F, Yousaf Z, Khan AA, Razok A, Akram J, Ali EAH, et al. SGLT-2 inhibitors associated euglycemic and hyperglycemic DKA in a multicentric cohort. *Sci Rep*. 2021;11(1):10293. doi:10.1038/s41598-021-89752-w

[52] Hussaini SA, Aziz A, Musa M, Alamin M, Danjuma M. Late-Onset Euglycemic Diabetic Ketoacidosis in a Patient With Massive Stroke Requiring Decompressive Craniectomy: A Case Report. *Cureus*. 2021;13(10):e18629-. doi:10.7759/cureus.18629

[53] Alsaleh H, Faleh MF, Salameh R, Elmalik A, Afzal MS, Chabalout MN, et al. Case report of empagliflozin-induced euglycemic diabetic ketoacidosis. *Journal of Emergency Medicine, Trauma and Acute Care*. 2023;2023(2):19. doi:https://dx.doi.org/10.5339/jemtac.2023.19

[54] Jang SW, Lee H. Euglycemic diabetic ketoacidosis following traumatic brain injury. *Am J Emerg Med*. 2024;77:232.e1-232.e3. doi:10.1016/j.ajem.2024.01.006

[55] Lee IH, Ahn DJ. Dapagliflozin-associated euglycemic diabetic ketoacidosis in a patient with type 2 diabetes mellitus: A case report. *Medicine (Baltimore)*. 2020;99(21):e20228-. doi:10.1097/MD.0000000000020228

[56] Jeon JY, Kim SK, Kim KS, Song SO, Yun JS, Kim BY, et al. Clinical characteristics of diabetic ketoacidosis in users and non-users of SGLT2 inhibitors. *Diabetes Metab*. 2019;45(5):453-457. doi:10.1016/j.diabet.2019.01.001

[57] Alabdaljabar MS, Abdullah KM, Almasood A, Ali SS, Ashmeg A. Euglycemic Diabetic Ketoacidosis in a Sedated Patient after Coronary Artery Bypass Grafting: A Case Report and Literature Review. *Case Rep Med*. 2021;2021:2086520. doi:10.1155/2021/2086520

[58] Altowayan WM. Empagliflozin induced euglycemic diabetic ketoacidosis. A case reports. *Ann Med Surg (Lond)*. 2022;84:104879. doi:10.1016/j.amsu.2022.104879

[59] Albugami MM, Ahmed M, Alobaid D. Empagliflozin-Induced Euglycemic Diabetic Ketoacidosis in Type 2 Diabetes Mellitus. *Saudi J Med Med Sci*. 2020;8(3):241-242. doi:10.4103/sjmms.sjmms_325_20

[60] Tan KT. Three cases of euglycaemic diabetic ketoacidosis related to the use of sodium-glucoseco-transporter-2 inhibitors and calorie restriction. *Singapore Med J*. 2021;62(3):153-154. doi:10.11622/SMEDJ.2021029

[61] Kim M kyung. Euglycemic diabetic ketoacidosis with SGLT2 inhibitors in lean type 2 diabetes. *Integr Obes Diabetes*. 2016;2(4).

[62] Chao HY, Kornelius E. Two alcoholic liver cirrhosis patients developed diabetic ketoacidosis after SGLT2 inhibitors-prescription. *J Formos Med Assoc*. 2020;119(12):1886-1887. doi:10.1016/j.jfma.2020.07.013

[63] Chan MJ, Weng CH, Hsu CW, Huang WH, Yen TH. Dapagliflozin associated ketoacidosis: A must know fact for nephrologists. *Nephrology (Carlton)*. 2018;23(2):192. doi:10.1111/nep.13039

[64] Chang YC, Leu MM, Lee CC, Zeng YH. SGLT2 Inhibitor-Associated Euglycemic Diabetic Ketoacidosis in Eight Cases in a Medical Center in Taiwan. *Formosan Journal of Endocrinology and Metabolism*. 2019;10:11-15.

[65] Pai MA, Wu TK, Chien SW, Chen CH, Kuo YC, Chen HP, et al. Euglycemic diabetic ketoacidosis associated with sodium–glucose cotransporter-2 inhibitors after surgery: A case report and review of literature. *Tungs’ Medical Journal*. 2023;17(2):85-88.

[66] Lin YH. Sodium-glucose cotransporter-2 inhibitors induced eu-glycemic diabetic ketoacidosis: The first report in a type 2 diabetic (T2D) Taiwanese and literature review of possible pathophysiology and contributing factors. *J Formos Med Assoc*. 2018;117(9):849-854. doi:10.1016/j.jfma.2018.02.004

[67] Wang CT, Chang CW, Lu YC, Lam HC, Ku SJ, Tseng KB, et al. An atypical hyperosmolar hyperglycemic state and diabetic ketoacidosis induced by sodium-glucose cotransporter-2 inhibitors: A case report. *J Formos Med Assoc*. 2020;119(8):1325-1328. doi:10.1016/j.jfma.2019.11.015

[68] Wong YC, Liu KL, Lee CL. Postoperative extremity gangrene in a patient with type 2 diabetes taking SGLT2 inhibitors: A case report. *Medicine (Baltimore)*. 2021;100(16):e25590-. doi:10.1097/MD.0000000000025590

[69] Thewjitcharoen Y, Wanothayaroj E, Tammawiwat C, Malidaeng A, Yenseung N, Nakasatien S, et al. A single center retrospective analysis of SGLT2i-associated diabetic ketoacidosis in Bangkok: The role of point-of-care blood ketone testing. *J Clin Transl Endocrinol Case Rep*. 2018;10:8-10. doi:10.1016/j.jecr.2018.08.001

[70] Güngörler N, Seyhan L, Pekkolay Z. Two cases of euglycemic diabetic ketoacidosis caused by dapagliflozin. *Dicle Tıp Dergisi*. 2022;49(2):385-389.

[71] Bombacı E, Cevik B, Haydarlar H, Saracoglu KT, Demirhan R. Critical Care Management of Diabetic Ketoacidosis Caused by Sodium-Glucose Co-Transporter 2 Inhibitor: A Case Report. *South Clin Istanb Eurasia*. 2020;31(3):287-288.

[72] Karakaya Z, Topal FE, Topal F, Payza U, Akyol PY. Euglisemic diabetic ketoacidotic coma caused by dapagliflozin. *Am J Emerg Med*. 2018;36(11):2136.e1-2136.e2. doi:10.1016/j.ajem.2018.08.054

[73] Baytuğan NZ, Çelik Aİ, Bezgin T, Çağdaş M. Euglycemic diabetic ketoacidosis associated with ST segment elevation myocardial infarction following SGLT-2 inhibitor therapy. *Am J Emerg Med*. 2023;71:250.e1-250.e3. doi:10.1016/j.ajem.2023.07.007

[74] Atay Can Kula MD, Hoca E, Ahbab S, Ataoglu HE. Sodium Glucose Cotransporter-2 (SGLT-2) Inhibitor Related Diabetic Ketoacidosis.

[75] Ozer O, Yorulmaz G. Euglycemic diabetic ketoacidosis associated with empagliflozin use in the course of the SARS-Cov-2 pandemic. *Journal of the College of Physicians and Surgeons Pakistan*. 2021;30(10):S110-S111. doi:https://dx.doi.org/10.29271/jcpsp.2020.Supp2.S110

[76] Mukthar M, Skaria S, Ahmed RAB, Elmalti AYM. Sodium-Glucose Co-Transporter 2 Inhibitor-induced Euglycemic Diabetic Ketoacidosis in a Type 2 Diabetes Patient Not Absolutely Insulin-deficient! *IBNOSINA JOURNAL OF MEDICINE AND BIOMEDICAL SCIENCES*. 2019;11(1):35-37. doi:10.4103/ijmbs.ijmbs_92_18

[77] Tannouri L, Gouda S, Abboud Y. A Case of Euglycemic Diabetic Ketoacidosis in Patient with Type 2 DM. *Open Journal of Emergency Medicine*. 2023;11(4):174-179.

[78] AlKindi F, Boobes Y, Shalwani F, Ansari J, Almazrouei R. Sodium-Glucose Cotransporter 2 Inhibitor (SGLT2i) Associated Diabetic Ketoacidosis in Oncology Patients: A Case Series and Literature Review. *Cureus*. 2024;16(2):e53816-. doi:10.7759/cureus.53816

[79] Almazrouei R, Alkindi F, Alshamsi A, Dawoud T, Chaaban A, Rahman MU. Severe Prolonged SGLT2i-induced Euglycemic Diabetic Ketoacidosis Refractory to Standard Therapy and Dialysis: Case Report and Literature Review. *Oman Med J*. 2022;37(3):e373-. doi:10.5001/omj.2022.17

[80] AlSaraj F, Krishnareddy K, Capatos G, Medhat P. EUGLYCEMIC DIABETIC KETOACIDOSIS IN A PATIENT WITH TYPE 2 DIABETES MELLITUS ON SODIUM-GLUCOSE COTRANSPORTER-2 INHIBITORS INDUCED BY COVID-19. *Medicine*. 2021;100(33).

[81] Kleinjan JP, Blom J, P van BA, Bouma HR, R van DP. Balancing Risks and Benefits: Sodium-Glucose Cotransporter 2 Inhibitors and the Risk of Diabetic Ketoacidosis. *Metabolites*. 2024;14(3). doi:10.3390/metabo14030162

[82] Wohlrab P, Kainz M, Schiferer A, Zapletal B, Tschernko E. Report Euglycemic Diabetic Ketoacidosis After Cardiac Surgery in a Patient Treated With Empagliflozin for Type 2 Diabetes Mellitus. *J Cardiothorac Vasc Anesth*. 2022;36(7):2066-2069. doi:10.1053/j.jvca.2021.07.048

[83] Oriot P, Hermans MP, Beauloye C, Rogghe PA, Noel S, Paternotte E. Unsuspected diabetic ketoacidosis after myocardial infarction in a patient treated with SGLT2 inhibitor increased length of stay in the hospital: how can it be prevented? A case report. *Eur Heart J Case Rep*. 2023;7(8):ytad336-. doi:10.1093/ehjcr/ytad336

[84] Menghoum N, Oriot P, Hermans MP. Clinical and biochemical characteristics and analysis of risk factors for euglycaemic diabetic ketoacidosis in type 2 diabetic individuals treated with SGLT2 inhibitors: A review of 72 cases over a 4.5-year period. *Diabetes & Metabolic Syndrome: Clinical Research & Reviews*. 2021;15(6):102275. doi:10.1016/j.dsx.2021.102275

[85] Sabbe M. Euglycemic Ketoacidosis and an Absence Sei-zure in A Type 2 Diabetic On SGLT2 Inhibitors: Case Report and Review of the Literature. *Ann Clin Med Case Rep*. 2021;8(3):1-5.

[86] Sitina M, Lukes M, Sramek V. Empagliflozin-associated postoperative mixed metabolic acidosis. Case report and review of pathogenesis. *BMC Endocr Disord*. 2023;23(1):81. doi:10.1186/s12902-023-01339-w

[87] Tougaard NH, Faber J, Eldrup E. Very low carbohydrate diet and SGLT-2-inhibitor: double jeopardy in relation to ketoacidosis. *BMJ Case Rep*. 2019;12(4). doi:10.1136/bcr-2018-227516

[88] Storgaard H, Bagger JI, Knop FK, Vilsbøll T, Rungby J. Diabetic Ketoacidosis in a Patient with Type 2 Diabetes After Initiation of Sodium-Glucose Cotransporter 2 Inhibitor Treatment. *Basic Clin Pharmacol Toxicol*. 2016;118(2):168-170. doi:10.1111/bcpt.12457

[89] Pilianidis G, Papanastasiou G, Tikoudi P, Themistocleous A, Farmakis G, Dolianitis K. Can euglycemic Diabetic Ketoacidosis Caused by SGLT2 Inhibitors be avoided in Covid-19 and other acute Infections? *Eur J Case Rep Intern Med*. 2024;11(3):4282. doi:10.12890/2024_004282

[90] Papadokostaki E, Liberopoulos E. Euglycemic Diabetic Ketoacidosis Secondary to Dapagliflozin in a Patient with Colon Malignancy. *Case Rep Endocrinol*. 2019;2019:3901741. doi:10.1155/2019/3901741

[91] Sampani E, Sarafidis P, Dimitriadis C, Kasimatis E, Daikidou D, Bantis K, et al. Severe euglycemic diabetic ketoacidosis of multifactorial etiology in a type 2 diabetic patient treated with empagliflozin: case report and literature review. *BMC Nephrol*. 2020;21(1):276. doi:10.1186/s12882-020-01930-6

[92] Papanastasiou L, Glycofridi S, Gravvanis C, Skarakis N, Papadimitriou I, Kanti G, et al. Diabetic ketoacidosis in patients treated with SGLT2 inhibitors: experience at a tertiary hospital. *Hormones (Athens)*. 2021;20(2):369-376. doi:10.1007/s42000-020-00256-0

[93] Rafey MF, Butt A, Coffey B, Reddington L, Devitt A, Lappin D, et al. Prolonged acidosis is a feature of SGLT2i-induced euglycaemic diabetic ketoacidosis. *Endocrinol Diabetes Metab Case Rep*. 2019;2019. doi:10.1530/EDM-19-0087

[94] Smyth CC, Collins M, McCarthy J, Galvin S. The catabolic triad: case report of fasting, major cardiac surgery and sodium-glucose co-transporter 2 inhibitors leading to perioperative euglycaemic ketoacidosis. *Interact Cardiovasc Thorac Surg*. 2021;33(3):494-495. doi:10.1093/icvts/ivab104

[95] Fahy Y, Laffey JG, McNicholas B. Sodium-Glucose Cotransporter 2 Inhibitor-induced Ketoacidosis and Non-Ketotic Acidosis: When Is There a Role for Bicarbonate? *Ann Am Thorac Soc*. 2023;20(6):911-916. doi:10.1513/AnnalsATS.202209-780CC

[96] Kumar D, Tudor R, Berzan E, Nasim MZ, Shah SA. Canagliflozin induced Diabetic Ketoacidosis. *Ir J Med Sci*. 2017;186:S372-S372.

[97] Bonora BM, Avogaro A, Fadini GP. Sodium-glucose co-transporter-2 inhibitors and diabetic ketoacidosis: An updated review of the literature. *Diabetes Obes Metab*. 2018;20(1):25-33. doi:10.1111/dom.13012

[98] Nappi F, A LV, Carfora G, Garofalo C, Provenzano M, Sasso FC, et al. Nephrology Consultation for Severe SGLT2 Inhibitor-Induced Ketoacidosis in Type 2 Diabetes: Case Report. *Medicina (Kaunas)*. 2019;55(8). doi:10.3390/medicina55080462

[99] Secinaro E, Ciavarella S, Rizzo G, Porreca E, Vitacolonna E. SGLT2-inhibitors and euglycemic diabetic ketoacidosis in COVID-19 pandemic era: a case report. *Acta Diabetol*. 2022;59(10):1391-1394. doi:10.1007/s00592-022-01909-9

[100] Burgio A, Alletto M, Amico S, Castiglione U, Fulco G, Groppuso C, et al. Euglycemic diabetic ketoacidosis in type 2 diabetes mellitus treated with sodium-glucose cotransporter 2 inhibitors. A report on two cases. *Italian Journal of Medicine*. 2019;13(1):54-58. doi:10.4081/itjm.2018.1061

[101] Kasbawala K, Stamatiades GA, Majumdar SK. Fournier’s Gangrene and Diabetic Ketoacidosis Associated with Sodium Glucose Co-Transporter 2 (SGLT2) Inhibitors: Life-Threatening Complications. *Am J Case Rep*. 2020;21:e921536-. doi:10.12659/AJCR.921536

[102] Mendonça FM, Silva MM, Chaves V, Souto S, Freitas P, Carvalho D. Diabetic Ketoacidosis after Bariatric Surgery: A Case Report. *REVISTA PORTUGUESA DE ENDOCRINOLOGIA DIABETES E METABOLISMO*. 2020;15(3-4):178-181. doi:10.26497/cc200047

[103] Calçada MB, Fernandes L, R SC, Montezinho S, F MD, Frutuoso L, et al. Euglycemic Diabetic Ketoacidosis after a Single Dose of Empagliflozin in a Patient with Pancreatitis. *Clin Pract*. 2021;11(2):216-218. doi:10.3390/clinpract11020031

[104] Fustiga J, Fernandes M, Dâmaso F, Duarte JA, Rodrigues C. Postop Complication With Euglycemic Diabetic Ketoacidosis in a Patient Receiving Empagliflozin. *Cureus*. 2022;14(12):e33161-. doi:10.7759/cureus.33161

[105] Ritchie DT, Dixon J. SGLT-2 inhibitor associated euglycaemic diabetic ketoacidosis in an orthopaedic trauma patient.*BMJ Case Rep*. 2022;15(9). doi:10.1136/bcr-2022-250233

[106] Richardson A, Vincent C. A case report of sodium glucose co-transporter 2 inhibitor associated euglycemic diabetic ketoacidosis: a diagnostic. *European Journal of Medical Case Reports*. 2022;6(1):11-16.

[107] Cavka L, U BF, Pitz N, Trpkovski Z, Lainscak M. Sodium-glucose cotransporter 2 inhibitor-induced euglycaemic diabetic ketoacidosis in heart failure with preserved ejection fraction. *ESC Heart Fail*. 2021;8(4):2631-2636. doi:10.1002/ehf2.13452

[108] Gil-Perdomo JM, F FGT, Jordán-Arias B, Domingo-Marín S, J GAJ, Martínez-Sagasti F. Diabetic ketoacidosis inducing myocardial infarction secondary to treatment with dapagliflozin: a case report. *Clin Case Rep*. 2017;5(6):809-811. doi:10.1002/ccr3.858

[109] Nzomessi D, Massie E, Gariani K, Giraud R, Meyer P. Combined lactic acidosis and ketoacidosis in a female diabetic patient with severe heart failure. *Cardiovasc Endocrinol Metab*. 2023;12(3):e0287-. doi:10.1097/XCE.0000000000000287

[110] Quarella M, Walser D, Brändle M, Fournier JY, Bilz S. Rapid Onset of Diabetic Ketoacidosis After SGLT2 Inhibition in a Patient With Unrecognized Acromegaly. *J Clin Endocrinol Metab*. 2017;102(5):1451-1453. doi:10.1210/jc.2017-00082

[111] Kietaibl AT, Fasching P, Glaser K, Petter-Puchner AH. New Diabetic Medication Sodium-Glucose Cotransporter-2 Inhibitors Can Induce Euglycemic Ketoacidosis and Mimic Surgical Diseases: A Case Report and Review of Literature. *Front Surg*. 2022;9:828649. doi:10.3389/fsurg.2022.828649

[112] Leader R, Cowen J, Rajeev SP. Dapagliflozin (SGLT2-i) induced euglycaemic diabetic ketoacidosis. *BMJ Case Rep*. 2019;12(9). doi:10.1136/bcr-2019-231104

[113] Sloan G, Kakoudaki T, Ranjan N. Prolonged diabetic ketoacidosis associated with canagliflozin. *Endocrinol Diabetes Metab Case Rep*. 2018;2018. doi:10.1530/EDM-17-0177

[114] Sanusi I, Sarnowski A, Russell-Jones D, Forni LG. A potential diagnostic problem on the ICU: Euglycaemic diabetic Ketoacidosis associated with SGLT2 inhibition. *J Crit Care*. 2020;57:19-22. doi:10.1016/j.jcrc.2019.12.007

[115] Kate W, Webb LA, Matthew F, Ketan D. Possible risk factors for the development of sodium-glucose co-transporter 2 inhibitor-associated diabetic ketoacidosis in type 2 diabetes. *British Journal of Diabetes and Vascular Disease*. 2016;16(2):78-81. doi:10.15277/bjd.2016.079

[116] Zac-Varghese S, Mathew S, Go D, Winocour P. A case series of diabetic ketoacidosis associated with SGLT2 inhibitors. *Practical Diabetes*. 2023;40(3):21-25a. doi:https://dx.doi.org/10.1002/pdi.2455

[117] Sleiwah A, McBride M, Black CE. Euglycaemic ketoacidosis: a potential new hazard to plastic surgery day case and inpatient procedures. *BMJ Case Rep*. 2017;2017. doi:10.1136/bcr-2017-220253

[118] Sharma M, Bandlamudi N, Idris I, Singhal R, Mahawar K, Madhok B. Sodium-Glucose Co-transporter-2 Inhibitors Induced Diabetic Ketoacidosis in Patients Undergoing Bariatric Surgery: a Systematic Review of Case Reports and Case Series. *Obes Surg*. Published online 2022.

[119] Vranić I, Stanković I, Panić M, Miličević P, Nešković AN. Empagliflozin-associated euglycemic ketoacidosis. *SRCEi krvni sudovi*.:186.

[120] Turner J, Begum T, Smalligan RD. Canagliflozin-Induced Diabetic Ketoacidosis: Case Report and Review of the Literature. *J Investig Med High Impact Case Rep*. 2016;4(3):2324709616663231-. doi:10.1177/2324709616663231

[121] Soni P, Kumar V, Saradna A, Kupfer Y. Empagliflozin-Associated Euglycemic Diabtetic Ketoacidosis. *Am J Ther*. 2018;25(6):e740-e741. doi:10.1097/MJT.0000000000000752

[122] Amianda EA, Gavigan TS, Talishinskiy T, Ewing DR, Schmidt HJ. Two cases of euglycemic diabetic ketoacidosis after bariatric surgery associated with sodium-glucose cotransporter-2 inhibitor use. *Obes Surg*. 2021;31(8):3848-3850. doi:10.1007/s11695-021-05391-0

[123] Iqbal I, Hamid M, Khan MAA, Kainat A, Tariq S. Dapagliflozin-induced Late-onset Euglycemic Diabetic Ketoacidosis. *Cureus*. 2019;11(11):e6089-. doi:10.7759/cureus.6089

[124] Gajjar K, Luthra P. Euglycemic Diabetic Ketoacidosis in the Setting of SGLT2 Inhibitor Use and Hypertriglyceridemia: A Case Report and Review of Literature. *Cureus*. 2019;11(4):e4384-. doi:10.7759/cureus.4384

[125] Bteich F, Daher G, Kapoor A, Charbek E, Kamel G. Post-surgical Euglycemic Diabetic Ketoacidosis in a Patient on Empagliflozin in the Intensive Care Unit. *Cureus*. 2019;11(4):e4496-. doi:10.7759/cureus.4496

[126] Candelario N, Wykretowicz J. The DKA that wasn’t: a case of euglycemic diabetic ketoacidosis due to empagliflozin. *Oxf Med Case Reports*. 2016;2016(7):144-146. doi:10.1093/omcr/omw061

[127] Chauhan S, Manov A, Dhillon GS, Shah P. Empagliflozin-Associated Euglycemic Diabetic Ketoacidosis in a Patient With Type 2 Diabetes Mellitus. *Cureus*. 2023;15(1):e33892-. doi:10.7759/cureus.33892

[128] Vitale RJ, Valtis YK, McDonnell ME, Palermo NE, Fisher NDL. Euglycemic Diabetic Ketoacidosis With COVID-19 Infection in Patients With Type 2 Diabetes Taking SGLT2 Inhibitors. *AACE Clin Case Rep*. 2021;7(1):10-13. doi:10.1016/j.aace.2020.11.019

[129] Acevedo-Mendez BA, Ye Y, Hajizadeh N, Myers A. Hypertriglyceridemia-Induced Acute Pancreatitis, Euglycemic Diabetic Ketoacidosis and COVID-19 Infection in a Patient With Type 2 Diabetes Taking a Sodium-Glucose Cotransporter 2 Inhibitor. *Cureus*. 2021;13(11):e19828-. doi:10.7759/cureus.19828

[130] Sexe J, Mayes C, Tofts P. Euglycemic Diabetic Ketoacidosis in a Lung Cancer Patient Using Empagliflozin. *Case Rep Crit Care*. 2020;2020:7437892. doi:10.1155/2020/7437892

[131] Chandrakumar HP, Chillumuntala S, Singh G, McFarlane SI. Postoperative Euglycemic Ketoacidosis in Type 2 Diabetes Associated with Sodium-Glucose Cotransporter 2 Inhibitor: Insights Into Pathogenesis and Management Strategy. *Cureus*. 2021;13(6):e15533-. doi:10.7759/cureus.15533

[132] Yehya A, Sadhu A. Sodium-Glucose Cotransporter 2 Inhibitor-Associated Prolonged Euglycemic Diabetic Ketoacidosis in Type 2 Diabetes: A Case Report and Literature Review. *Clin Diabetes*. 2020;38(1):112-116. doi:10.2337/cd19-0035

[133] Chai PR, Bonney C, Blohm E, Boyer EW, Babu KM. Canagliflozin-associated diabetic ketoacidosis: a case report. *Toxicol Commun*. 2017;1(1):2-5. doi:10.1080/24734306.2017.1331604

[134] Pongracz B, Theodore D, Titus B, Dube K. Euglycemic ketoacidosis in a postoperative deformity correction spine surgery patient: A case report. *Crit Care Med*. 2019;47(1 Supplement 1).

[135] Ahmed T, Karimi H, Hegde V, Lodhi SH. Euglycemic Diabetic Ketoacidosis Due to SGLT2 Inhibitor in a Patient With Gitelman Syndrome: A Therapeutic Dilemma. *Cureus*. 2021;13(10):e19169-. doi:10.7759/cureus.19169

[136] Diaz-Ramos A, Eilbert W, Marquez D. Euglycemic diabetic ketoacidosis associated with sodium-glucose cotransporter-2 inhibitor use: a case report and review of the literature. *Int J Emerg Med*. 2019;12(1):27. doi:10.1186/s12245-019-0240-0

[137] Osafehinti DA, Okoli OJ, Karam JG. A Case of SGLT2 Inhibitor-Associated Euglycemic Diabetic Ketoacidosis Following Coronary Artery Bypass Surgery. *AACE Clin Case Rep*. 2021;7(1):20-22. doi:10.1016/j.aace.2020.11.014

[138] Tauseef A, Asghar MS, Zafar M, Lateef N, Thirumalareddy J. Sodium-glucose linked transporter inhibitors as a cause of euglycemic diabetic ketoacidosis on a background of starvation. *Cureus*. 2020;12(8).

[139] Badwal K, Tariq T, Peirce D. Dapagliflozin-Associated Euglycemic Diabetic Ketoacidosis in a Patient Presenting with Acute Pancreatitis. *Case Rep Endocrinol*. 2018;2018:6450563. doi:10.1155/2018/6450563

[140] Garay PS, Zuniga G, Lichtenberg R. A Case of Euglycemic Diabetic Ketoacidosis Triggered by a Ketogenic Diet in a Patient With Type 2 Diabetes Using a Sodium-Glucose Cotransporter 2 Inhibitor. *Clin Diabetes*. 2020;38(2):204-207. doi:10.2337/cd19-0055

[141] Peters AL, Buschur EO, Buse JB, Cohan P, Diner JC, Hirsch IB. Euglycemic Diabetic Ketoacidosis: A Potential Complication of Treatment With Sodium-Glucose Cotransporter 2 Inhibition. *Diabetes Care*. 2015;38(9):1687-1693. doi:10.2337/dc15-0843

[142] Rao SJ, Kumar K, Saleh N. A Case of SGLT2 Inhibitor-Induced Euglycemic Diabetic Ketoacidosis. *Cureus*. 2022;14(10):e30106-. doi:10.7759/cureus.30106

[143] Fang J, Genco M, Caskey RN. COVID-19 Precipitating Euglycaemic Diabetic Ketoacidosis with SGLT2 Inhibitor Use. *Eur J Case Rep Intern Med*. 2020;7(11):1943. doi:10.12890/2020_001943

[144] Khedr A, Hennawi HA, Khan MK, Eissa A, Mir M, Rauf I, et al. Sodium-glucose cotransporter-2 inhibitor-associated euglycemic diabetic ketoacidosis in COVID-19-infected patients: A systematic review of case reports. *World J Clin Cases*. 2023;11(24):5700-5709. doi:10.12998/wjcc.v11.i24.5700

[145] Erondu N, Desai M, Ways K, Meininger G. Diabetic Ketoacidosis and Related Events in the Canagliflozin Type 2 Diabetes Clinical Program. *Diabetes Care*. 2015;38(9):1680-1686. doi:10.2337/dc15-1251

[146] Haddadin R, Aboujamra D, Iraninezhad H. Sodium-Glucose Cotransporter-2 Inhibitor-Induced Euglycemic Diabetic Ketoacidosis in a Type 2 Diabetic Patient. *Cureus*. 2023;15(12):e51184-. doi:10.7759/cureus.51184

[147] Gorlitsky B, El-Ibiary S. A Common but Not so Typical Elevated Anion Gap. *American Journal of Medicine*. 2016;129(5):e25-e26. doi:10.1016/j.amjmed.2015.12.021

[148] Bell-McClure E, Sabol V, McMillan K. Complicated Acidosis Presentations: When Is Diabetic Ketoacidosis Not Diabetic Ketoacidosis? A Case Series. *Journal for Nurse Practitioners*. 2023;19(3). doi:10.1016/j.nurpra.2022.11.011

[149] Singh B, Kaur P, Majachani N, Patel P, Reid RJR, Maroules M. COVID-19 and Combined Diabetic Ketoacidosis and Hyperglycemic Hyperosmolar Nonketotic Coma: Report of 11 Cases. *J Investig Med High Impact Case Rep*. 2021;9. doi:10.1177/23247096211021231

[150] Tirthani E, Said M, Neupane B, Quartuccio M. An Unusual Case of the “Terrible Triad” in a Transgender Woman. *Cureus*. 2021;13(8):e16869-. doi:10.7759/cureus.16869

[151] Allison R, Goldstein D, Musso MW. Challenges in the Diagnosis of Euglycemic Diabetic Ketoacidosis in a Patient With Multiple Sclerosis Taking a Sodium-Glucose Cotransporter 2 Inhibitor. *J Emerg Med*. 2019;57(1):e1-e3. doi:10.1016/j.jemermed.2019.03.011

[152] Seger CD, Xing H, Wang L, Shin JS. Intraoperative Diagnosis of Sodium-Glucose Cotransporter 2 Inhibitor-Associated Euglycemic Diabetic Ketoacidosis: A Case Report. *A A Pract*. 2021;15(1):e01380-. doi:10.1213/XAA.0000000000001380

[153] Wojtas C, Rasarmos AP, Naddaf N. Sodium-Glucose Transport Protein 2 Inhibitors Association with Euglycemic Diabetic Ketoacidosis. *Case Rep Endocrinol*. 2023;2023:6835882. doi:10.1155/2023/6835882

[154] Smith A, Holtrop J, Sadoun M. Post-operative euglycemic diabetic ketoacidosis in a patient with SGLT-2 inhibitor use and recent sleeve gastrectomy. *Cureus*. 2021;13(4).

[155] Latif A, Gastelum AA, Sood A, Reddy JT. Euglycaemic diabetic ketoacidosis in a 43-year-old woman with type 2 diabetes mellitus on SGLT-2 inhibitor (empagliflozin). *BMJ Case Rep*. 2020;13(6). doi:10.1136/bcr-2020-235117

[156] Gammons DT, Counselman FL. Sodium-glucose Cotransporter-2 Induced Diabetic Ketoacidosis with Minimal Hyperglycemia. *Clin Pract Cases Emerg Med*. 2018;2(1):47-50. doi:10.5811/cpcem.2017.12.36213

[157] Dyatlova N, Omotosho YB, Sherchan R, Shrestha J, Buddharaju V. A Case of Severe Metabolic Acidosis due to Jardiance-Induced Euglycemic Diabetic Ketoacidosis. *Cureus*. 2021;13(4):e14580-. doi:10.7759/cureus.14580

[158] Strawn KD, Davis KW. The Interconnectedness of Euglycemic Diabetic Ketoacidosis With Concomitant Thyroid Storm: A Case Report. *Cureus*. 2024;16(4):e58696-. doi:10.7759/cureus.58696

[159] Tiwari K, Sharma NR, Pokhrel M, Basnet A, Kaplan M. Misleading Presentation: Chest Pain Masking Euglycemic Diabetic Ketoacidosis Possibly Induced by Empagliflozin. *Cureus*. 2023;15(11):e49402-. doi:10.7759/cureus.49402

[160] Kelmenson DA, Burr K, Azhar Y, Reynolds P, Baker CA, Rasouli N. Euglycemic Diabetic Ketoacidosis With Prolonged Glucosuria Associated With the Sodium-Glucose Cotransporter-2 Canagliflozin. *J Investig Med High Impact Case Rep*. 2017;5(2):2324709617712736-. doi:10.1177/2324709617712736

[161] Latif A, Gastelum AA, Sood A, Reddy JT. Euglycaemic diabetic ketoacidosis in a 43-year-old woman with type 2 diabetes mellitus on SGLT-2 inhibitor (empagliflozin). *Drug Ther Bull*. 2021;59(6):93-95. doi:10.1136/dtb.2021.235117rep

[162] Auerbach JS, Gershengorn HB, Aljure OD, Lamelas J, Patel SS, Ferreira TD, et al. Postcardiac Surgery Euglycemic Diabetic Ketoacidosis in Patients on Sodium-Glucose Cotransporter 2 Inhibitors. *J Cardiothorac Vasc Anesth*. 2023;37(6):956-963. doi:10.1053/j.jvca.2023.01.041

[163] Voskertchian A, Milanes L. Case Report: DKA with Lower-than-Expected Blood Glucose in the Setting of SGLT2 Inhibitor Use. *Am Fam Physician*. 2021;103(4):200.

[164] Koch RA, Clark RF. Euglycemic Ketoacidosis With Sodium-Glucose Cotransporter-2 Inhibitor. *Am J Ther*. 2018;25(5):e590-e591. doi:10.1097/MJT.0000000000000602

[165] Aggarwal A, Jain A, Sachdeva S, Kulairi ZI. Prolonged Glucosuria With Sodium-Glucose Cotransporter-2 (SGLT2) Inhibitors: A Case Report and Review of Literature. *Cureus*. 2020;12(11):e11554-. doi:10.7759/cureus.11554

[166] Stamatiades GA, D’Silva P, Elahee M, Viana GM, Sideri-Gugger A, Majumdar SK. Diabetic Ketoacidosis Associated with Sodium-Glucose Cotransporter 2 Inhibitors: Clinical and Biochemical Characteristics of 29 Cases. *Int J Endocrinol*. 2023;2023:6615624. doi:10.1155/2023/6615624

[167] Zughaib MT, Patel K, Leka M, Affas S. Self-Induced Euglycemic Diabetic Ketoacidosis: When to Stop the Drip. *Cureus*. 2022;14(1):e21768-. doi:10.7759/cureus.21768

[168] Dull RB, Spangler ML, Knezevich EL, Lau BM. Euglycemic Diabetic Ketoacidosis Associated With Sodium-Glucose Cotransporter Type 2 Inhibitors in Patients With Type 2 Diabetes Mellitus Receiving Oral Therapy. *J Pharm Pract*. 2019;32(2):240-243. doi:10.1177/0897190017748049

[169] Mendelsohn RA, Taveras AN, Mazer BA. Euglycemic Diabetic Ketoacidosis Precipitated by SGLT-2 Inhibitor Use, Pericarditis, and. *Clinical Practice and Cases in Emergency Medicine, 4 (3)*. Published online 2020.

[170] Rathore A, Gupta N, Kahn C, Kadariya D. Euglycemic diabetic ketoacidosis caused by empagliflozin complicated by failure to thrive in a geriatric patient. *Arch Clin Cases*. 2023;10(2):89-92. doi:10.22551/2023.39.1002.10248

[171] Andrews TJ, Cox RD, Parker C, Kolb J. Euglycemic Diabetic Ketoacidosis with Elevated Acetone in a Patient Taking a Sodium-Glucose Cotransporter-2 (SGLT2) Inhibitor. *J Emerg Med*. 2017;52(2):223-226. doi:10.1016/j.jemermed.2016.07.082

[172] Puls HA, Haas NL, Franklin BJ, Theyyunni N, Harvey CE. Euglycemic diabetic ketoacidosis associated with SGLT2i use: Case series. *Am J Emerg Med*. 2021;44:11-13. doi:10.1016/j.ajem.2021.01.033

[173] Zughaib M, Basharat B, Small D. A Case of STEMI-Induced Euglycemic Diabetic Ketoacidosis in a Patient Receiving a Sodium Glucose Cotransporter-2 Inhibitor. *JACC Case Rep*. 2023;11:101792. doi:10.1016/j.jaccas.2023.101792

[174] Petersen C, Gyabaah F, Sotelo J, Yohanna S, Deoker A. A Case of Prolonged Recovery for Post-percutaneous Coronary Intervention (PCI) Sodium-Glucose Cotransporter-2 (SGLT2) Inhibitor-Induced Euglycemic Diabetic Ketoacidosis in a 28-Year-Old. *Cureus*. 2023;15(9):e45180-. doi:10.7759/cureus.45180

[175] Fieger EI, Fadel KM, Modarres AH, 3rd WEP, Wolver SE. SUCCESSFUL REIMPLEMENTATION OF A VERY LOW CARBOHYDRATE KETOGENIC DIET AFTER SGLT2 INHIBITOR ASSOCIATED EUGLYCEMIC DIABETIC KETOACIDOSIS. *AACE Clin Case Rep*. 2020;6(6):e330-e333. doi:10.4158/ACCR-2020-0314

[176] Ullah S, Khan N, Zeb H, Tahir H. Metabolic ketoacidosis with normal blood glucose: A rare complication of sodium-glucose cotransporter 2 inhibitors. *SAGE Open Med Case Rep*. 2016;4:2050313X16675259-. doi:10.1177/2050313X16675259

[177] Khalid MM, Vearrier DJ, Greenberg MI. Euglycemic ketoacidosis following a single dose of empagliflozin. *Toxicol Commun*. 2017;1(1):18-20.

[178] Roach P, Skierczynski P. Euglycemic Diabetic Ketoacidosis in a Patient With Type 2 Diabetes After Treatment With Empagliflozin. *Diabetes Care*. 2016;39(1):e3-. doi:10.2337/dc15-1797

[179] Kapila V, Topf J. Sodium-Glucose Co-transporter 2 Inhibitor-Associated Euglycemic Diabetic Ketoacidosis After Bariatric Surgery: A Case and Literature Review. *Cureus*. 2021;13(8):e17093-. doi:10.7759/cureus.17093

[180] Arlas N, Vandiver JW. Fanconi syndrome and euglycemic diabetic ketoacidosis secondary to canagliflozin use in a type 2 diabetic. *J Clin Transl Endocrinol Case Rep*. 2022;23((Arlas, Vandiver) Saint Joseph Family Medicine Residency Program, United States(Vandiver) University of Wyoming School of Pharmacy, United States):100109. doi:https://dx.doi.org/10.1016/j.jecr.2022.100109

[181] Pace DJ, Dukleska K, Phillips S, Gleason V, Yeo CJ. Euglycemic Diabetic Ketoacidosis Due to Sodium-Glucose Cotransporter 2 Inhibitor Use in Two Patients Undergoing Pancreatectomy. *J Pancreat Cancer*. 2018;4(1):95-99. doi:10.1089/pancan.2018.0016

[182] Dass B, Beck A, Holmes C, Morton G. Euglycemic DKA (euDKA) as a presentation of COVID-19. *Clin Case Rep*. 2021;9(1):395-398. doi:10.1002/ccr3.3540

[183] Shoukat S, Usmani NA, Soetan O, Qureshi F. Euglycemic Diabetic Ketoacidosis Accompanied by Severe Hypophosphatemia During Recovery in a Patient With Type 2 Diabetes Being Treated With Canagliflozin/Metformin Combination Therapy. *Clin Diabetes*. 2017;35(4):249-251. doi:10.2337/cd16-0027

[184] Steinmetz-Wood S, Gilbert M, Menson K. A Case of Diabetic Ketoacidosis in a Patient on an SGLT2 Inhibitor and a Ketogenic Diet: A Critical Trio Not to Be Missed. *Case Rep Endocrinol*. 2020;2020:8832833. doi:10.1155/2020/8832833

[185] A FFD, G ML, Sharma S, E AMC. A Rare Case of Empagliflozin-Induced Euglycemic Diabetic Ketoacidosis Obscured by Alkalosis. *Cureus*. 2022;14(6):e25818-. doi:10.7759/cureus.25818

[186] Khan M, Khalid S, Marwat A, Mehmood H. A case of euglycemic diabetic ketoacidosis due to canagliflozin complicated by Takotsubo cardiomyopathy. *Am J Med Case Rep*. 2018;6:1-3.

[187] C van N, Wallace J, Takata M, Yu R. Euglycaemic diabetic ketoacidosis in bariatric surgery patients with type 2 diabetes taking canagliflozin. *BMJ Case Rep*. 2018;2018. doi:10.1136/bcr-2017-221527

[188] Guirguis H, S BA, Pham C. The Use of SGLT-2 Inhibitors Coupled With a Strict Low-Carbohydrate Diet: A Set-Up for Inducing Severe Diabetic Ketoacidosis. *Clin Med Insights Case Rep*. 2022;15:11795476221090044-. doi:10.1177/11795476221090045

[189] A MML, Ahmed S, M SRA, Poretsky L. Hyperglycemic DKA in a patient with type 2 diabetes mellitus on monotherapy with SGLT-2 inhibitor. *J Clin Transl Endocrinol Case Rep*. 2024;32((Medina Mora, Ahmed, Sanchez Ruiz, Poretsky) Division of Endocrinology, Diabetes, and Metabolism, Northwell Health, Lenox Hill Hospital, 110 E 59th Street, Suite 8B, New York, NY 10022, United States):100166. doi:https://dx.doi.org/10.1016/j.jecr.2024.100166

[190] Wang KM, Isom RT. SGLT2 Inhibitor-Induced Euglycemic Diabetic Ketoacidosis: A Case Report. *Kidney Med*. 2020;2(2):218-221. doi:10.1016/j.xkme.2019.12.006

[191] Lindsay PJ, Gibson LE, Bittner EA, Berg S, Chang MG. Sodium-glucose cotransporter-2 (SGLT2) inhibitor-induced euglycemic diabetic ketoacidosis complicating the perioperative management of a patient with type 2 diabetes mellitus (T2DM) and Fournier’s gangrene: A case report. *Int J Surg Case Rep*. 2020;77:463-466. doi:10.1016/j.ijscr.2020.11.037

[192] Earle M, Ault B, Bonney C. Euglycemic Diabetic Ketoacidosis in Concurrent Very Low-carbohydrate Diet and Sodium-glucose Transporter-2 Inhibitor Use: A Case Report. *Clin Pract Cases Emerg Med*. 2020;4(2):185-188. doi:10.5811/cpcem.2020.2.45904

[193] Kum-Njii JLS, Gosmanovi AR, Steinbergi HO, Dagogo-Jack S. Hyperglycemic, high anion-gap metabolic acidosis in three patients receiving SGLT-2 inhibitors for diabetes management. *Endocr Rev*. 2016;37(2 Supplement 1). doi:https://dx.doi.org/10.1210/endo-meetings.2016.DGM.6.SUN-750

[194] Bonanni FB, Fei P, Fitzpatrick LL. Normoglycemic ketoacidosis in a postoperative gastric bypass patient taking canagliflozin. *Surg Obes Relat Dis*. 2016;12(1):e11-2. doi:10.1016/j.soard.2015.08.502

[195] Gupta S, Mohta A, Temitope S. Life-Threatening Acidosis With Metformin and Dapagliflozin Combination Therapy: A Case Report. *Cureus*. 2023;15(2):e35497-. doi:10.7759/cureus.35497

[196] Wasey W, Hutchings S, Dufner A, Okon D, Saleh S. Fall, Fracture, and Two Episodes of Euglycemic Diabetic Ketoacidosis. *Cureus*. 2022;14(6):e25788-. doi:10.7759/cureus.25788

[197] Iqbal QZ, Mishiyev D, Niazi MR, Zia Z, Sattar SBA, Jahanghir A, et al. SGLT-2 Inhibitors-a Culprit of Diabetic Ketoacidosis Postbariatric Surgery. *Case Rep Crit Care*. 2020;2020:8817829. doi:10.1155/2020/8817829

[198] Darwish AM. Metabolic Acidosis in Postsurgical Patient on Canagliflozin and Metformin: A Case Report. *A A Pract*. 2019;12(7):221-222. doi:10.1213/XAA.0000000000000888

[199] Westcott GP, Segal AR, Mitri J, Brown FM. Prolonged glucosuria and relapse of diabetic ketoacidosis related to SGLT2-inhibitor therapy. *Endocrinol Diabetes Metab*. 2020;3(2):e00117-. doi:10.1002/edm2.117

[200] Danford C, Chan P, Magill SB. ‘Euglycemic’ Ketoacidosis in a Patient With Type 2 Diabetes Being Treated With Canagliflozin. *WMJ*. 2016;115(4):206-209.

[201] Benmoussa JA, Clarke M, Penmetsa A, Khine LY, Leykina L, Diaz K, et al. Euglycemic diabetic ketoacidosis: The clinical concern of SGLT2 inhibitors. *J Clin Transl Endocrinol Case Rep*. 2016;2:17-19. doi:10.1016/j.jecr.2016.05.002

[202] Elshimy G, Joy CM, Scander S, Gibiezaite S. Case report of sudden onset hypoglycemia post fluid resuscitation in diabetic ketoacidosis in patient with type 2 diabetes on dapagliflozin without prior insulin use. *Endocr Rev*. 2018;39(2 Supplement 1).

[203] Morrison N, Barnett K, Tantum J, Morrison HK, Whalen M. A case of euglycemic diabetic ketoacidosis in a patient with type 2 diabetes mellitus and COVID-19. *Cureus*. 2020;12(12).

[204] Misaghian-Xanthos N, Shariff AI, Mekala K, Fearrington LR, Setji TL, Aloi JA, et al. Sodium-Glucose Cotransporter 2 Inhibitors and Diabetic Ketoacidosis: A Case Series From Three Academic Institutions. *Diabetes Care*. 2017;40(6):e65-e66. doi:10.2337/dc16-2591

[205] Tito E, Ramaswami A, Milbocker R, Edmond D. Euglycemic Diabetic Ketoacidosis Presenting as Hypoglycemia in a Patient With Type 2 Diabetes and Von Gierke’s Disease. *Cureus*. 2024;16(1):e52104-. doi:10.7759/cureus.52104

[206] Kang CY, Khamooshi P, V RP. An Unsuspected Case of Euglycemic Diabetic Ketoacidosis With Twists. *Cureus*. 2022;14(4):e24016-. doi:10.7759/cureus.24016

[207] Chauhan S, Manov A, Shah P. Euglycemic Ketoacidosis Secondary Due to SGLT2 Inhibitors. Published online 2023.

[208] Mistry S, Eschler DC. Euglycemic Diabetic Ketoacidosis Caused by SGLT2 Inhibitors and a Ketogenic Diet: A Case Series and Review of Literature. *AACE Clin Case Rep*. 2021;7(1):17-19. doi:10.1016/j.aace.2020.11.009

[209] Sood M, Simon B, Ryan KF, Zebrower M. Euglycemic Diabetic Ketoacidosis with SGLT2 Inhibitor Use in A Patient on The Atkins Diet: A Unique Presentation of A Known Side Effect. *AACE Clin Case Rep*. 2018;4(2):104-107. doi:10.4158/EP171860.CR

[210] VonTungeln CD, M AB. Euglycemic Diabetic Ketoacidosis Induced by Sodium-Glucose Cotransporter-2 Inhibitor Use and Coronary Angiography: A Case Report. *Cureus*. 2024;16(1):e52122-. doi:10.7759/cureus.52122

[211] Sabanci R, Saaed M, Bandi A, Das K, Wilcox M. Beyond the Benefits: A Case Study on the Complications of Sodium-Glucose Co-Transporter-2 (SGLT2) Inhibitors (Euglycemic Diabetic Ketoacidosis (DKA) and Takotsubo Cardiomyopathy). *Cureus*. 2024;16(2):e55068-. doi:10.7759/cureus.55068

[212] Pujara S, Ioachimescu A. Prolonged Ketosis in a Patient With Euglycemic Diabetic Ketoacidosis Secondary to Dapagliflozin. *J Investig Med High Impact Case Rep*. 2017;5(2):2324709617710040-. doi:10.1177/2324709617710040

[213] Mehta PB, Robinson A, Burkhardt D, Rushakoff RJ. Inpatient Perioperative Euglycemic Diabetic Ketoacidosis Due to Sodium-Glucose Cotransporter-2 Inhibitors - Lessons From a Case Series and Strategies to Decrease Incidence. *Endocr Pract*. 2022;28(9):884-888. doi:10.1016/j.eprac.2022.06.006

[214] Bardhi O, Bloom MD, Sattari M. Euglycaemic diabetic ketoacidosis in a patient with pancreatitis and type 2 diabetes on empagliflozin. *BMJ Case Rep*. 2022;15(6). doi:10.1136/bcr-2021-247921

[215] Clark A, Mohammed AS, Raut A, Moore S, Houlden R, Awad S. Prevalence and Clinical Characteristics of Adults Presenting With Sodium-Glucose Cotransporter-2 Inhibitor-Associated Diabetic Ketoacidosis at a Canadian Academic Tertiary Care Hospital. *Can J Diabetes*. 2021;45(3):214-219. doi:10.1016/j.jcjd.2020.08.100

[216] Shamchuk A, Doulla M, Jetha M. Cases: Possible association between diabetic ketoacidosis and use of sodium-glucose co-transporter 2 inhibitor in a 17-year-old youth with type 2 diabetes. *CMAJ*. 2021;193(35):E1385-E1388. doi:10.1503/cmaj.202627

[217] Lau A, Bruce S, Wang E, Ree R, Rondi K, Chau A. Perioperative implications of sodium-glucose cotransporter-2 inhibitors: a case series of euglycemic diabetic ketoacidosis in three patients after cardiac surgery. *Can J Anaesth*. 2018;65(2):188-193. doi:10.1007/s12630-017-1018-6

[218] Alexander LD, Yu C. Diabetic Ketoacidosis in A Patient with Type 2 Diabetes On Canagliflozin and Dexamethasone. *AACE Clin Case Rep*. 2017;3(4):331-335. doi:10.4158/EP161506.CR

[219] Chaudhry A, Roels C, Lee J. Sodium-Glucose Cotransporter-2 Inhibitor-associated Euglycemic Diabetic Ketoacidosis: Lessons From a Case Series of 4 Patients Undergoing Coronary Artery Bypass Grafting Surgery. *Can J Diabetes*. 2022;46(8):843-850. doi:10.1016/j.jcjd.2022.06.007

[220] Clement M, Senior P. Euglycemic diabetic ketoacidosis with canagliflozin *Not*-*so*-*sweet but avoidable complication of sodium*-*glucose cotransporter*-*2 inhibitor use*. *CANADIAN FAMILY PHYSICIAN*. 2016;62(9):725-728.

[221] Dizon S, Keely EJ, Malcolm J, Arnaout A. Insights Into the Recognition and Management of SGLT2-Inhibitor-Associated Ketoacidosis: It’s Not Just Euglycemic Diabetic Ketoacidosis. *Can J Diabetes*. 2017;41(5):499-503. doi:10.1016/j.jcjd.2017.05.004

[222] Jazi M, Porfiris G. Euglycemic diabetic ketoacidosis in type 2 diabetes treated with a sodium-glucose cotransporter-2 inhibitor. *Can Fam Physician*. 2016;62(9):722-724.

[223] Leung M, Rodrigues P, Roitman D. Ketoacidosis in a Patient with Type 2 Diabetes Requiring Alpelisib: Learnings and Observations Regarding Alpelisib Initiation and Rechallenge. *Onco Targets Ther*. 2022;15:1309-1315. doi:10.2147/OTT.S370244

[224] Zhang L, Tamilia M. Euglycemic diabetic ketoacidosis associated with the use of a sodium-glucose cotransporter-2 inhibitor. *CMAJ*. 2018;190(25):E766-E768. doi:10.1503/cmaj.171319

[225] Wood T, Pang AJ, Hallet J, Greig P. Euglycaemic ketoacidosis in a postoperative Whipple patient using canaglifozin. *BMJ Case Rep*. 2016;2016. doi:10.1136/bcr-2016-216607

[226] Lane S, Paskar D, Hamed S, Goffi A. When Guidelines Fail: Euglycemic Diabetic Ketoacidosis After Bariatric Surgery in a Patient Taking a Sodium-Glucose Cotransporter-2 Inhibitor: A Case Report. *A A Pract*. 2018;11(2):46-48. doi:10.1213/XAA.0000000000000734

[227] Jazi M, Porfiris G. Euglycemic diabetic ketoacidosis in type 2 diabetes treated with a sodium-glucose cotransporter-2 inhibitor (vol 62, 722, 2016). *CANADIAN FAMILY PHYSICIAN*. 2016;62(11):877.

[228] Banakh I, Kung R, Gupta S, Matthiesson K, Tiruvoipati R. Euglycemic diabetic ketoacidosis in association with dapagliflozin use after gastric sleeve surgery in a patient with type II diabetes mellitus. *Clin Case Rep*. 2019;7(5):1087-1090. doi:10.1002/ccr3.2147

[229] Hawkins AM, Jackson R V, White H, Vardesh DL. SGLT2-inhibitor induced euglycemic ketoacidosis in acute surgical patients. *Journal of Case Reports and Images in Surgery*. 2017;3:41-46.

[230] Thiruvenkatarajan V, Meyer EJ, Nanjappa N, M VWR, Jesudason D. Perioperative diabetic ketoacidosis associated with sodium-glucose co-transporter-2 inhibitors: a systematic review. *Br J Anaesth*. 2019;123(1):27-36. doi:10.1016/j.bja.2019.03.028

[231] Umapathysivam MM, Morgan B, Inglis JM, Meyer E, Liew D, Thiruvenkatarajan V, et al. SGLT2 Inhibitor–Associated Ketoacidosis vs Type 1 Diabetes–Associated Ketoacidosis. *JAMA Netw Open*. 2024;7(3):e242744-e242744.

[232] Meyer EJ, Mignone E, Hade A, Thiruvenkatarajan V, Bryant R V, Jesudason D. Periprocedural Euglycemic Diabetic Ketoacidosis Associated With Sodium-Glucose Cotransporter 2 Inhibitor Therapy During Colonoscopy. *Diabetes Care*. 2020;43(11):e181-e184. doi:10.2337/dc20-1244

[233] Hamblin PS, Wong R, Ekinci EI, Fourlanos S, Shah S, Jones AR, et al. SGLT2 Inhibitors Increase the Risk of Diabetic Ketoacidosis Developing in the Community and During Hospital Admission. *J Clin Endocrinol Metab*. 2019;104(8):3077-3087. doi:10.1210/jc.2019-00139

[234] Lee S, Morgan A, Shah S, Ebeling PR. Rapid-onset diabetic ketoacidosis secondary to nivolumab therapy. *Endocrinol Diabetes Metab Case Rep*. 2018;2018. doi:10.1530/EDM-18-0021

[235] O’Neill RS, Tyack L, Freeman M, Hussein HS. Euglycemic Ketoacidosis in a Patient with Metastatic Non-Small-Cell Lung Adenocarcinoma and Concomitant Pulmonary Embolism. *Case Rep Endocrinol*. 2020;2020:8882299. doi:10.1155/2020/8882299

[236] McCann M, O’Brien A, Larbalestier R, Davis T. Sodium-glucose cotransport-2 inhibitor induced ketoacidosis following coronary artery bypass surgery: implications for management. *Intern Med J*. 2022;52(5):876-879. doi:10.1111/imj.15772

[237] Chacko B, Whitley M, Beckmann U, Murray K, Rowley M. Postoperative euglycaemic diabetic ketoacidosis associated with sodium-glucose cotransporter-2 inhibitors (gliflozins): a report of two cases and review of the literature. *Anaesth Intensive Care*. 2018;46(2):215-219. doi:10.1177/0310057X1804600212

[238] Meyer EJ, Gabb G, Jesudason D. SGLT2 inhibitor-associated euglycemic diabetic ketoacidosis: A South Australian clinical case series and Australian spontaneous adverse event notifications. *Diabetes Care*. 2018;41(4):e47-e49. doi:10.2337/dc17-1721

[239] Ahn T, Teloken P, Burke P. Sodium-glucose linked transporter 2 inhibitor associated post-operative euglycaemic diabetic ketoacidosis: an important consideration for all surgeons. *ANZ J Surg*. 2019;89(7-8):982. doi:10.1111/ans.15307

[240] Thiruvenkatarajan V, Nanjappa N, Sembu M, Meyer EJ, M VWR, Jesudason D. An analysis of the Australian Therapeutic Goods Administration Database of Adverse Event Notifications of diabetic ketoacidosis associated with sodium-glucose cotransporter-2 inhibitors in surgical patients. *Anaesth Intensive Care*. 2020;48(1):70-72. doi:10.1177/0310057X19892988

[241] O’Brolchain A, Maletsky J, Mian I, Edwards S. Does Treatment with Sodium-Glucose Cotransporter-2 Inhibitors Affect Adherence to International Society Criteria for Diabetic Ketoacidosis in Adult Patients with Type 2 Diabetes? A Retrospective Cohort Analysis. *J Diabetes Res*. 2024;2024:1-8. doi:10.1155/2024/1849522

[242] Jhaveri U, Vardesh D. Sodium-glucose Cotransporter-2 Inhibitors and Euglycaemic Diabetic Ketoacidosis in the Perioperative Period: Case Report. *Cureus*. 2019;11(8):e5455-. doi:10.7759/cureus.5455

[243] Isaacs M, Tonks KT, Greenfield JR. Euglycaemic diabetic ketoacidosis in patients using sodium-glucose co-transporter 2 inhibitors. *Intern Med J*. 2017;47(6):701-704. doi:10.1111/imj.13442

[244] Lucero P, Chapela S. Euglycemic Diabetic Ketoacidosis in the ICU: 3 Case Reports and Review of Literature. *Case Rep Crit Care*. 2018;2018:1747850. doi:10.1155/2018/1747850

[245] Batista D V, CAFA V, Costa TA, Lima EG. COVID-19-associated euglycemic diabetic ketoacidosis in a patient with type 2 diabetes on SGLT2 inhibitor: a case report. *Diabetol Int*. 2021;12(3):313-316. doi:10.1007/s13340-020-00473-3

[246] Pontes JPJ, de Melo CS, Arantes FBB, de Souza Ramos JTG, Módolo NSP, Navarro e Lima LH. Perioperative euglycemic diabetic ketoacidosis following use of SGLT-2 inhibitors after cardiac surgery. *J Clin Anesth*. 2021;71. doi:10.1016/j.jclinane.2021.110201

[247] Jimenez-Montero JG. Diabetic Ketoacidosis Linked with Sodium Glucose Co-Transporter 2 Inhibitors in an Elderly Patient with Type 2 Diabetes. *Int J Endocrinol Metab Disord*. 2019;5(1).

**Supplementary Material 5:** Precipitants of DKA in cases reported within the meta-summary

| **DKA precipitants** | **Total population**  **(Studies [s]=247, [n]=1024)** | **Asia**^1–80^  **(Studies [s]=80, [n]=269)** | **Europe**^81–119^  **(Studies [s]=39, [n]=146)** | **North America**^120–227^  **(Studies [s]=108, [n]=299)** | **Oceania**^228–243^  **(Studies [s]=16, [n]=305)** | **South America**^244–247^ **(Studies [s]=4, [n]=5)** |
| --- | --- | --- | --- | --- | --- | --- |
| Alcohol related (%, n) | 0.2% (n=2) | 0.4% (n=1) | 0.0% (n=0) | 0.3% (n=1) | 0.0% (n=0) | 0.0% (n=0) |
| COVID (%, n) | 2.4% (n=25) | 0.7% (n=2) | 1.4% (n=2) | 6.7% (n=20) | 0.0% (n=0) | 20.0% (n=1) |
| Reduced oral intake | 5.2% (n=53) | 4.8% (n=13) | 4.0% (n=6) | 9.4% (n=28) | 1.6% (n=5) | 20.0% (n=1) |
| Ketogenic/ Low carbohydrate diet | 1.7% (n=17) | 1.1% (n=3) | 1.4% (n=2) | 3.3% (n=10) | 0.7% (n=2) | 0.0% (n=0) |
| Sepsis (%, n) | 5.4% (n=55) | 5.2% (n=14) | 4.8% (n=7) | 10.0% (n=30) | 1.3% (n=4) | 0.0% (n=0) |
| Suboptimal adherence to diabetes management (%, n) | 2.1% (n=21) | 4.5% (n=12) | 0.0% (n=0) | 2.7% (n=8) | 0.3% (n=1) | 0.0% (n=0) |
| Surgical admission (%, n) | 14.9% (n=153) | 12.6% (n=34) | 4.8% (n=7) | 14.7% (n=44) | 21.6% (n=66) | 40.0% (n=2) |
| Unknown/Not recorded (%, n) | 22.3% (n=228) | 22.7% (n=61) | 15.8% (n=23) | 19.7% (n=59) | 27.9% (n=85) | 0.0% (n=0) |
| SGLT2i related (%, n) | 37.3% (n=382) | 23.0% (n=62) | 66.4% (n=97) | 27.8% (n=83) | 45.6% (n=139) | 20.0% (n=1) |
| Acute kidney injury (%, n) | 0.5% (n=5) | 0.4% (n=1) | 0.0% (n=0) | 0.7% (n=2) | 0.7% (n=2) | 0.0% (n=0) |
| Stroke (%, n) | 0.3% (n=3) | 0.4% (n=1) | 0.0% (n=0) | 0.7% (n=2) | 0.0% (n=0) | 0.0% (n=0) |
| Myocardial infarction (%, n) | 2.5% (n=26) | 6.7% (n=18) | 0.7% (n=1) | 2.0% (n=6) | 0.3% (n=1) | 0.0% (n=0) |
| Pancreatitis (%, n) | 5.0% (n=52) | 16.7% (n=45) | 0.7% (n=1) | 2.0% (n=6) | 0.0% (n=0) | 0.0% (n=0) |
| Liver cirrhosis (%, n) | 0.2% (n=2) | 0.7% (n=2) | 0.0% (n=0) | 0.0% (n=0) | 0.0% (n=0) | 0.0% (n=0) |

**Supplementary Material 6:** Baseline characteristics of DKA cases from DEKODE database – unmatched population

| **Parameter** | **Overall [n=1060]** | **SGLT2i non-users [n=793]** | **SGLT2i users [n=267]** | **p-value** | **SGLT2i (with precipitating cause) [n=186]** | **SGLT2i (related)**  **[n=81]** | **p-value** |
| --- | --- | --- | --- | --- | --- | --- | --- |
| Gender (Female), [%, n] | 44.7% (n=474) | 44.1% (n=350) | 46.4% (n=124) | .559 | 41.9% (n=78) | 56.8% (n=46) | .035 |
| Age (mean (SD)), [years] | 62.9 (14.2) | 63.5 (14.7) | 61.1 (12.7) | .020 | 61.7 (12.3) | 59.7 (13.6) | .239 |
| Ethnicity (%) |  | | | .006 |  | | .486 |
| *Asian* | 14.2% (n=151) | 15.6% (n=124) | 10.1% (n=27) |  | 11.8% (n=22) | 6.2% (n=5) |  |
| *Black* | 7.0% (n=74) | 7.7% (n=61) | 4.9% (n=13) |  | 4.8% (n=9) | 4.9% (n=4) |  |
| *Unknown* | 8.4% (n=89) | 9.3% (n=74) | 5.6% (n=15) |  | 5.4% (n=10) | 6.2% (n=5) |  |
| *Other* | 3.1% (n=33) | 3.2% (n=25) | 3.0% (n=8) |  | 3.8% (n=6) | 1.2% (n=1) |  |
| *White* | 67.3% (n=713) | 64.2% (n=509) | 76.4% (n=204) |  | 74.2% (n=138) | 81.5% (n=66) |  |
| BMI (mean (SD)) [kg m²] | 30.0 (10.7) | 30.1 (11.1) | 29.6 (9.4) | .557 | 29.8 (9.7) | 29.3 (8.9) | .694 |
| Charlson Co-morbidity Index (CCI),  [mean (SD)] |  | | | .095 |  | | .173 |
| <3 points | 12.1% (n=128) | 10.8% (n=86) | 15.7% (n=42) |  | 15.1% (n=28) | 17.3% (n=14) |  |
| >3 points | 25.5% (n=270) | 25.5% (n=202) | 25.5% (n=68) |  | 22.6% (n=42) | 32.1% (n=26) |  |
| Unknown | 62.5% (n=662) | 63.7% (n=505) | 58.8% (n=157) |  | 62.4% (n=116) | 50.6% (n=41) |  |

BMI: Body Mass Index, CCI: Charlson Co-morbidity Index.

**Supplementary Material 7:** Differences in DKA precipitants between SGLT2i users vs SGLT2i non-users; non-SGLT2i users vs SGLT2i (with precipitating cause) and SGLT2i-related DKA vs SGLT2i (with precipitating cause) – matched for Age, Ethnicity, BMI, CCI.

| **Parameter** | **Overall [n=534]** | **SGLT2i non-users [n=267]** | **SGLT2i users [n=267]** | **p-value** | **SGLT2i non-users [n=186]** | **SGLT2i (with precipitating cause) [n=186]** | **p-value** |
| --- | --- | --- | --- | --- | --- | --- | --- |
| Alcohol related (%, n) | 1.3% (n=7) | 1.9% (n=5) | 0.7% (n=2) | .447 | 4.3% (n=8) | 1.1% (n=2) | .109 |
| COVID (%, n) | 4.3% (n=23) | 3.4% (n=9) | 5.2% (n=14) | .394 | 5.4% (n=10) | 7.5% (n=14) | .527 |
| Drug-induced (%, n) | 2.1% (n=11) | 1.9% (n=5) | 2.2% (n=6) | 1.00 | 2.7% (n=5) | 3.2% (n=6) | 1.000 |
| Intercurrent illness (%, n) | 39.1% (n=209) | 43.4% (n=116) | 34.8% (n=93) | .051 | 43.0% (n=80) | 50.0% (n=93) | .212 |
| Sepsis (%, n) | 11.0% (n=59) | 11.6% (n=31) | 10.5% (n=28) | .782 | 12.4% (n=23) | 15.1% (n=28) | .547 |
| Suboptimal adherence to diabetes management (%, n) | 16.5% (n=88) | 21.3% (n=57) | 11.6% (n=31) | .004 | 19.9% (n=37) | 16.7% (n=31) | .502 |
| Reclassified to type 2 diabetes | 3.6% (n=19) | 7.1% (n=19) | - | - | 5.9% (n=11) | - | - |
| Surgical admission (%, n) | 0.6% (n=3) | 1.1% (n=3) | 0.0% (n=0) | .247 | 0.0% (n=0) | 0.0% (n=0) | - |
| Unknown (%, n) | 6.4% (n=34) | 8.2% (n=22) | 4.5% (n=12) | .111 | 6.5% (n=12) | 6.5% (n=12) | 1.000 |
| SGLT2i related (%, n) | - | - | 30.3% (n=81) | - | - | - | - |

**Supplementary Material 8:** Differences in management parameters of DKA cases from DEKODE database between SGLT2i users vs SGLT2i non-users; non-SGLT2i users vs SGLT2i (with precipitating cause) and SGLT2i-related DKA vs SGLT2i (with precipitating cause) – matched for age, gender, BMI, ethnicity, CCI, precipitating cause and admission biochemistry. FRIII: fixed rate intravenous insulin infusion; Perc: percentage.

| **Parameter** | **Overall [n=534]** | **SGLT2i non-users**  **[n=267]** | **SGLT2i users**  **[n=267]** | **p-value** | **SGLT2i non-users [n=267]** | **SGLT2i (with precipitating cause) [n=81]** | **p-value** | **SGLT2i (with precipitating cause) [n=81]** | **SGLT2i (related)**  **[n=81]** | **p-value** |
| --- | --- | --- | --- | --- | --- | --- | --- | --- | --- | --- |
| FRIII rate (units/hour) [mean (SD)] | 6.9 (3.1) | 7.2 (3.3) | 6.6 (2.8) | .040 | 7.2 (3.3) | 6.6 (2.8) | .080 | 5.9 (3.2) | 6.7 (2.8) | .114 |
| FRIII appropriateness (%)[mean (SD)] | 93.5 (21.2) | 93.3 (19.3) | 93.7 (23.0) | .835 | 91.9 (19.9) | 93.7 (24.5) | .415 | 97.2 (26.8) | 93.7 (19.5) | .346 |
| Estimated total intravenous insulin volume  (units) [mean (SD)] | 144.2 (165.0) | 151.7 (195.5) | 136.8 (127.4) | .298 | 155.4 (210.8) | 136.7 (125.7) | .299 | 114.1 (110.7) | 137.0 (132.0) | .233 |
| Perc Fluids (%) [mean (SD)] | 93.4 (50.0) | 91.6 (50.6) | 95.2 (49.4) | .408 | 90.5 (50.3) | 99.8 (53.1) | .082 | 99.6 (51.3) | 84.5 (37.5) | .034 |
| Perc Glucose (%) [mean (SD)] | 94.6 (38.6) | 96.8 (40.7) | 92.3 (36.4) | .173 | 95.2 (39.0) | 92.7 (35.3) | .511 | 94.6 (36.9) | 91.3 (39.0) | .585 |
| Perc Ketones (%) [mean (SD)] | 69.6 (33.4) | 68.7 (34.4) | 70.4 (32.5) | .570 | 68.1 (35.9) | 72.2 (33.4) | .260 | 78.2 (36.6) | 66.2 (30.0) | .023 |
